# WDR23 regulates NRF2 independently of KEAP1

**DOI:** 10.1371/journal.pgen.1006762

**Published:** 2017-04-28

**Authors:** Jacqueline Y. Lo, Brett N. Spatola, Sean P. Curran

**Affiliations:** 1 University of Southern California, Leonard Davis School of Gerontology, Los Angeles, California, United States of America; 2 University of Southern California, Dornsife College of Letters, Arts, and Sciences, Department of Molecular and Computational Biology, Los Angeles, California, United States of America; Swiss Institute for Experimental Cancer Research, SWITZERLAND

## Abstract

Cellular adaptation to stress is essential to ensure organismal survival. NRF2/NFE2L2 is a key determinant of xenobiotic stress responses, and loss of negative regulation by the KEAP1-CUL3 proteasome system is implicated in several chemo- and radiation-resistant cancers. Advantageously using *C*. *elegans* alongside human cell culture models, we establish a new WDR23-DDB1-CUL4 regulatory axis for NRF2 activity that operates independently of the canonical KEAP1-CUL3 system. WDR23 binds the DIDLID sequence within the Neh2 domain of NRF2 to regulate its stability; this regulation is not dependent on the KEAP1-binding DLG or ETGE motifs. The C-terminal domain of WDR23 is highly conserved and involved in regulation of NRF2 by the DDB1-CUL4 complex. The addition of WDR23 increases cellular sensitivity to cytotoxic chemotherapeutic drugs and suppresses NRF2 in KEAP1-negative cancer cell lines. Together, our results identify WDR23 as an alternative regulator of NRF2 proteostasis and uncover a cellular pathway that regulates NRF2 activity and capacity for cytoprotection independently of KEAP1.

## Author summary

Chronic exposure to environmental stressors throughout life (“the exposome”) has been tied to several cancers in humans. Cellular adaptation to stress is essential to ensure organismal survival, and NRF2 is an exceptionally well-studied and key determinant of cellular stress responses that plays complex roles in cancer biology and responses to xenobiotics, including chemotherapies. Our studies have established a functional and evolutionarily conserved role for WDR23 as a substrate receptor for the Cullin4(CUL4)-DDB1 E3-ubiquitin ligase, which regulates NRF2 protein levels and activity, and which operates independently of the canonical KEAP1-CUL3 pathway. KEAP1 has been the most highly studied regulator of NRF2, as mutations in KEAP1, which result in uncontrolled activation of NRF2 and chemo-resistance, are found in many aggressive cancers. Importantly, increased expression of WDR23 in KEAP1(-/-) cancer cells restores aberrant NRF2 regulation. In the absence of a KEAP1-like system, *C*. *elegans* WDR-23 has been shown to regulate the worm cytoprotective transcription factor SKN-1. We have leveraged *C*. *elegans* genetic approaches to identify conserved regulatory mechanisms of mammalian cytoprotection by NRF2. Collectively, our studies suggest control of NRF2 homeostasis is much more sophisticated than previously appreciated.

## Introduction

In response to environmental and cellular stress, organisms must activate specific pathways to defend and protect against damage[1–3]. Such stressors include electrophiles, pathogens, and xenobiotics, many of which are carcinogens and activate the conserved cap-n-collar transcription factor NRF2 (nuclear factor E2-related factor) stress response pathway[2, 4]. In the presence of such stress, negative regulation of NRF2 is relieved, which leads to accumulation in the nucleus. Upon activation, NRF2 regulates the expression of genes with antioxidant response elements (ARE) in their promoters[5–7]. Activation of NRF2 cytoprotection pathways has been functionally linked to longevity[2, 8, 9], but when left unchecked, can be detrimental[10] and enhance cancer severity and resistance to chemotherapy[11].

The regulation of NRF2 is of particular importance to the progression of human diseases where oxidative stress plays a mechanistic role, including: cancer[12], inflammation[13], neurodegeneration[14], cardiovascular diseases[15], and even wound repair and regeneration[16]. In humans, the CUL3 (Cullin 3) and KEAP1 (Kelch-like ECH-associated protein 1) E3 ubiquitin ligase complex maintains NRF2 at low levels[17, 18]. KEAP1 is a bric-a-brac, tramtrack, broad complex (BTB) domain-containing protein that when bound to NRF2, facilitates polyubiquitination and degradation by the 26S proteasome[19]. However, recent studies allude to additional, but unidentified, layers of regulation that are independent of KEAP1[20].

In *C*. *elegans*, a mechanistically similar pathway negatively regulates the abundance of SKN-1, the worm equivalent of NRF2, but *via* the action of WDR-23[21, 22] and the CUL-4 E3 ubiquitin ligase, not CUL-3[23]. WDR-23 is a WD40-repeat protein, containing seven repeats of the tryptophan aspartic acid (WD) containing motif. This structure facilitates protein-protein interactions, and in particular, WD40 proteins have been shown to interact with the CUL4-DDB1 (damaged DNA binding protein 1) E3 ubiquitin ligase complex[24]. In worms, the CUL4-DDB1 ubiquitin ligase complex has been shown to associate with WDR-23, and together, they suppress expression of oxidative stress genes through regulation of SKN-1[21]. In the absence of *wdr-23*, SKN-1 is able to translocate into the nucleus, where it is able to serve as the transcription factor responsible for turning on oxidative stress genes leading to increased stress resistance[25–32] and lifespan extension[21, 33].

Surprisingly, the similarities between KEAP1 and worm WDR-23 are only mechanistic, as KEAP1 is structurally dissimilar to WDR-23. Despite the presence of KEAP1, the human genome has retained WDR23—also referred to as the DDB1 and CUL4 Associated Factor 11 (DCAF11) protein. Here we demonstrate functional regulation of the NRF2 cytoprotection pathway by the CUL4-DDB1-WDR23 ubiquitin proteasome system as an alternate to the canonical KEAP1 regulatory pathway. This finding is of great importance as loss of the KEAP1-dependent regulation of NRF2 is prevalent in several cancers that are hallmarked by resistance to chemo- and radiation- therapies, a side effect of NRF2-dependent activation of cytoprotection pathways.

## Results

### Human WDR23 regulates activation of NRF2 cytoprotection pathways

**Fig 1:**
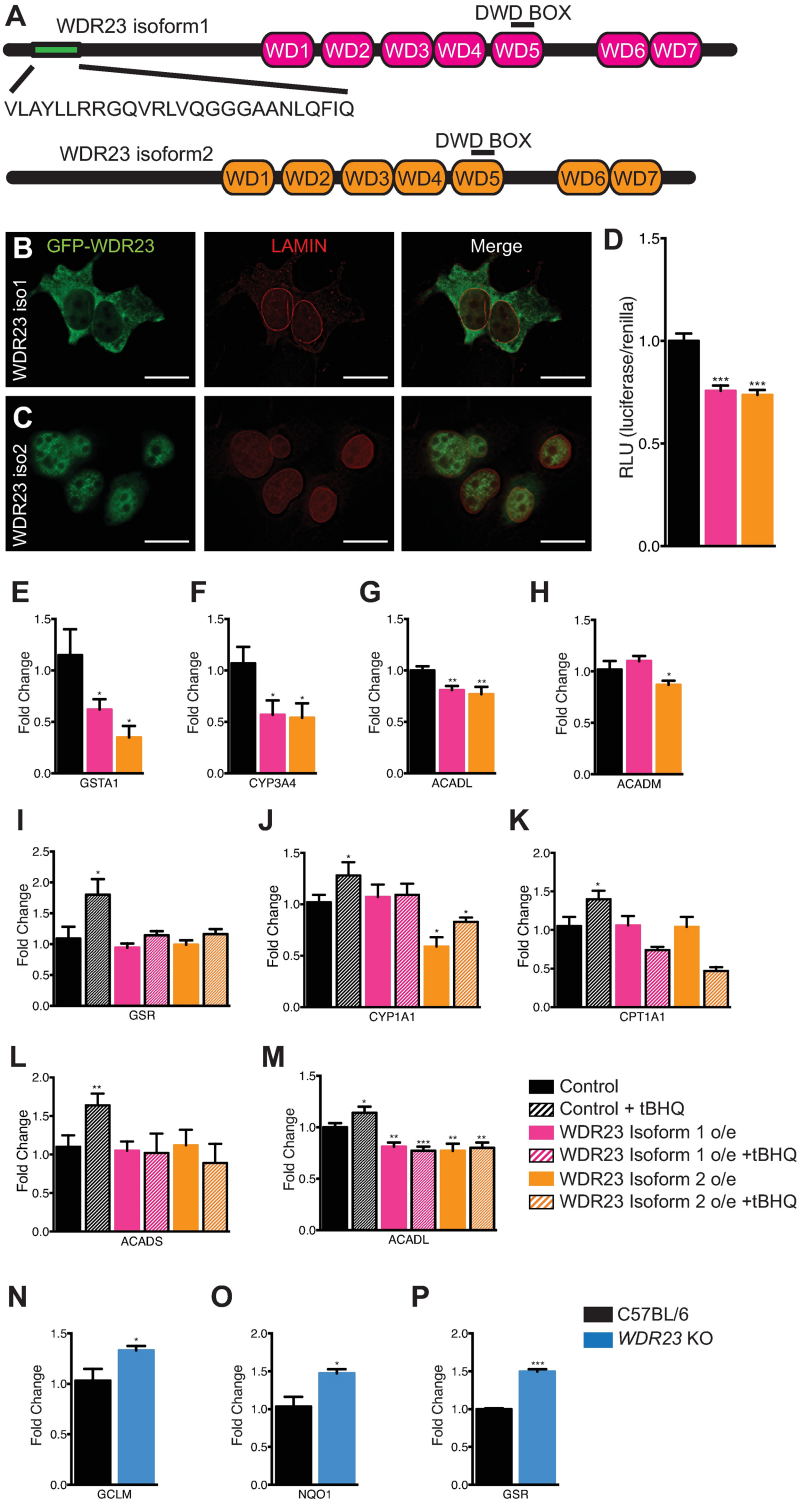
WDR23 influences cytoprotective pathways via regulation of NRF2. (**A**) Schematic of the domains found in human WDR23 isoform 1 (pink) and WDR23 isoform 2 (orange). (**B,C**) Representative images of HEK-293T cells overexpressing mCherry:LAMIN and either GFP:WDR23 isoform 1 (**B**) or GFP:WDR23 isoform 2 (**C**). Scale bar, 20μm. (**D**) Overexpression of WDR23 isoform 1 or WDR23 isoform 2 reduces the expression of an ARE-inducible luciferase reporter as compared to control (GFP expression) (Control n = 32, Iso 1 n = 24, Iso 2 n = 24). (**E-H**) Cells expressing WDR23 have reduced levels of the NRF2 target genes *GSTA1* (Control n = 9, Iso 1 n = 6, Iso 2 n = 3) (**E**), *CYP3A4* (Control n = 9, Iso 1 n = 6, Iso 2 n = 3) (**F**), *ACADL* (Control n = 12, Iso 1 n = 9, Iso 2 n = 9) (**G**), and *ACADM* (Control n = 12, Iso 1 n = 9, Iso 2 n = 9) (**H**) as compared to control (GFP expression). (**I-M**) Overexpression of WDR23 abrogates the effects of tBHQ-treatment on NRF2 targets *GSR* (Control n = 9, +tBHQ n = 9, Iso 1 n = 6, Iso 1 +tBHQ n = 6, Iso 2 n = 3, Iso 2 +tBHQ n = 3) (**I**), *CYP1A1* (Control n = 9, +tBHQ n = 9, Iso 1 n = 6, Iso 1 +tBHQ n = 6, Iso 2 n = 3, Iso 2 +tBHQ n = 3) (**J**), *CPT1A1* (Control n = 12, +tBHQ n = 12, Iso 1 n = 9, Iso 1 +tBHQ n = 9, Iso 2 n = 9, Iso 2 +tBHQ n = 9) (**K**), *ACADS* (Control n = 12, +tBHQ n = 12, Iso 1 n = 9, Iso 1 +tBHQ n = 9, Iso 2 n = 9, Iso 2 +tBHQ n = 9) (**L**), and *ACADL* (Control n = 12, +tBHQ n = 12, Iso 1 n = 9, Iso 1 +tBHQ n = 9, Iso 2 n = 9, Iso 2 +tBHQ n = 9) (**M**) as compared to control (GFP overexpression). *Wdr23* knockout MEF cells have increased levels of the NRF2 target genes *Gclm* (Control n = 5, KO n = 12) (**N**), *Nqo1* (Control n = 5, KO n = 12) (**O**), and *Gsr* (Control n = 5, KO n = 12) (**P**). Data are mean ± s.e.m.; (**D**) two-tailed *t*-test relative to control samples; (**E-P**) one-tailed *t*-test relative to control samples. *P<0.05, **P<0.01, ***P<0.001.

WDR-23 is the major regulator of SKN-1 activity, which is the *C*. *elegans* equivalent to mammalian NRF2/NFE2L2. Nematodes lack a KEAP1 homolog, but WDR-23 regulation of SKN-1 is mechanistically similar to KEAP1 regulation of NRF2, regulating turnover of the transcription factor by the ubiquitin proteasome system. Despite the evolution of the KEAP1 regulatory pathway, the WDR23 locus is exceptionally well conserved from worms to humans (Fig 1A, S1A Fig). Remarkably, a role for WDR23 in the regulation of the NRF2 cytoprotection pathway has yet to be described, and a general understanding of the role WDR23 plays in cell biology is lacking; there are two studies that have demonstrated a role for WDR23 in the regulation of SLBP[34, 35], and the only other published report describes altered expression of WDR23/DCAF11 in the mouse bladder epithelium in response to increased levels of urea and nitric oxide[36]. However, WDR23 has been identified in association with the CUL4-DDB1 E3 ligase complex, but like most E3 ligase receptors, specific target substrates remain elusive[37].

Two major isoforms (iso) of WDR23 are expressed in mammals (Fig 1A). WDR23 isoform 1 (UniProtKB/Swiss-Pro Accession: Q8TEB1-2) encodes a 546 amino acid polypeptide with a predicted molecular mass of 61.7 kDa, while the second isoform, WDR23 isoform 2 (UniProtKB/Swiss-Prot Accession: Q8TEB1-1), encodes a 520 amino acid polypeptide with a predicted molecular mass of 58.8 kDa. GFP tagged WDR23 isoform 1 is localized primarily to the cytoplasm (Fig 1B), while GFP:WDR23 isoform 2 is enriched in the nucleus, but can be found in the cytoplasm when overexpressed in HEK-293T (Fig 1C) or HepG2 (S1B and S1C Fig) cells. The cellular distribution of the two isoforms in human cell culture is consistent with the localization of the two predominant *Ce*WDR-23 isoforms in worms (S1D and S1E Fig)[38]. Although NRF2 activation by xenobiotic electrophiles leads to NRF2 accumulation in the nucleus[39], the subcellular localization of WDR23 does not change with stress (S1F–S1I Fig). The localization of WDR23 isoform 2 in the nucleus is intriguing, as KEAP1 regulation of NRF2 is thought to be restricted to the cytoplasm[40–42]. As such, KEAP1 and WDR23 may coordinately regulate NRF2 in either compartment.

To mount an appropriate response to cellular stress, NRF2 regulates the expression of several classes of xenobiotic response genes, including: glutathione homeostasis, drug metabolism, iron metabolism, multidrug resistance transporters, cellular energy metabolism, biogenesis of circulatory signaling molecules and receptors, and calcium homeostasis[43]. These genes all contain an antioxidant response element (ARE) and are positively regulated by NRF2. To assess whether WDR23 is a functional regulator of NRF2 cytoprotection pathways, we measured NRF2-dependent activation of an ARE-luciferase reporter co-transfected with a renilla control plasmid in HEK-293T cells that were overexpressing GFP tagged WDR23 (Fig 1D). ARE-luciferase activity was inversely related to WDR23 expression levels, supporting a model where WDR23 functions as a negative regulator of NRF2. Surprisingly, expression of *Ce*WDR-23 did not impact ARE-luciferase expression in unstressed cells or in KEAP1 siRNA treated cells. Thus, although WDR23 is an ancient regulator of cytoprotection, its functionality in the SKN-1 and NRF2 pathways is species specific (S2A and S2B Fig).

We were intrigued by the ability of WDR23 to influence the expression of cellular antioxidant responses *via* the ARE. We next determined if WDR23 could repress the expression of specific NRF2 targets that are responsible for the diversity in NRF2 cellular stress response [27, 44–50]. Increased expression of WDR23 resulted in reduced steady state expression of several NRF2 targets, including: *GSTA1* (Fig 1E), *CYP3A4* (Fig 1F), *ACADL* (Fig 1G), and *ACADM* (Fig 1H); however, not all NRF2 targets were altered (S2C–S2E Fig). Turning off the NRF2 response is equally important, particularly in the context of cancer cells where NRF2 is deregulated. Treatment of cells with *tert*-butylhydroquinone (tBHQ) activates NRF2-dependent transcription of cytoprotection genes[39], but when combined with WDR23 overexpression, the induction of electrophile induced NRF2 targets, including: *GSR* (Fig 1I), *CYP1A1* (Fig 1J), *CPT1A1* (Fig 1K), *ACADS* (Fig 1L), and *ACADL* (Fig 1M) were attenuated, while other NRF2-dependent transcripts were unaffected (S2F–S2H Fig). The fact that not all NRF2 targets were influenced by WDR23 may be indicative of the WDR23-regulatory pathway to direct a specific subset of NRF2 targets, of the differential impact the WDR23-NRF2 pathway plays in NRF2-cytoprotection in a cell-type dependent manner, or perhaps one function of the WDR23 control is to turn off NRF2 following transcriptional activation, which is more important for some targets.

In order to determine the effects of loss of WDR23, we derived MEF cells from the *Wdr23* knockout (KO) mouse that we generated. *Wdr23* KO MEF cells behave similarly to wildtype MEF cells, and we have not observed any differences in cellular fitness between the two genotypes. In line with the overexpression data that we had observed, the MEF KO cells show the opposite effect and have increased expression of NRF2 target genes, including: *Gclm* (Fig 1N), *Nqo1* (Fig 1O), and *Gsr* (Fig 1P). Notably, these cells have functional KEAP1; in fact, there is an increase in *Keap1* transcript levels (S2J Fig), consistent with the model of KEAP1 and WDR23 behaving complementary to each other. Additionally, the changes in NRF2 activity from modulation of WDR23 levels are independent of the phenotypes associated with WDR23’s role in SLBP regulation, as we do not observe an increase in NRF2 target expression when cells are depleted of *Slbp* (S2K–S2Q Fig). Unlike the WDR23 studies about SLBP by Brodersen et. al., reducing WDR23 levels in this context does not appear to be pleiotropic, likely due to compensation from KEAP1. Together with the overexpression data, these results demonstrate WDR23’s role as a negative regulator of NRF2.

### CUL4-DDB-1-WDR23 regulates NRF2 stability

**Fig 2:**
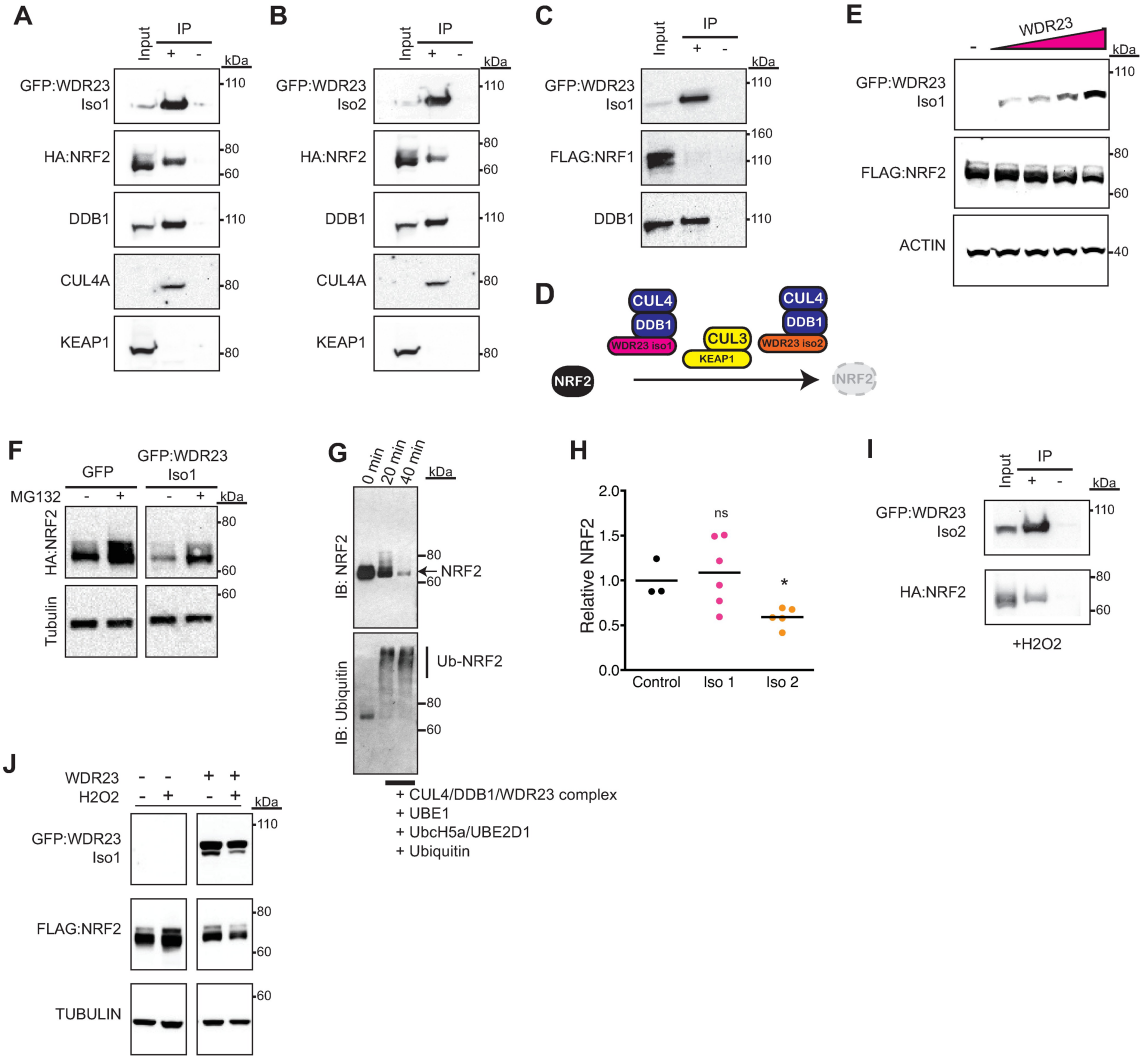
CUL4-DDB1-WDR23 regulates NRF2 stability. (**A-D**) In cells overexpressing tagged versions of WDR23 and NRF2, NRF2 immunoprecipitates (IP) with WDR23 isoform 1 (**A**) or WDR23 isoform 2 (**B**) along with DDB1 and CUL4A, but not KEAP1. NRF1 does not IP with the WDR23-DDB1 complex in cells overexpressing tagged versions of WDR23 and NRF1 (**C**); as diagramed in (**D**). (**E-H**) overexpressing WDR23 reduces the abundance of co-expressed FLAG:NRF2 (**E**) in a proteasome dependent manner (**F**), increases the abundance of poly-ubiquitinated-NRF2 in a time dependent manner (**G**), and can also reduce endogenous NRF2 in total cell lysate (**H**) as compared to control (GFP expression). Control n = 3, Iso 1 n = 6, Iso 2 n = 5. (**I**) WDR23 interacts with NRF2 in cells treated with H2O2 to induce oxidative stress. (**J**) The increased stability of NRF2 following H2O2 exposure is abrogated when WDR23 is overexpressed. Oxidative stress also destabilizes WDR23. *P<0.05.

The CUL4-DDB1 E3 ligase complex licenses WDR proteins as receptors for substrate recognition; however, very few receptor-substrate pairs are defined. *Ce*WDR-23 is thought to physically bind SKN-1[21] to regulate its abundance in the cell, but an interaction between WDR23 and NRF2 in humans has not been shown. To reveal the ability of WDR23 to interact with NRF2, we transfected cells with GFP:WDR23 and HA-NRF2 and tested for an interaction biochemically. We immunoprecipitated (IP) GFP:WDR23 isoform 1 (Fig 2A) or GFP:WDR23 isoform 2 (Fig 2B) and found that HA-NRF2 was efficiently co-immunoprecipitated with either isoform of WDR23, which indicates the ability of these two proteins to complex. Importantly, overexpression of GFP alone however did not sequester NRF2 (S3A Fig).

We were able to co-IP the several components of the CUL4 complex. IP of WDR23 efficiently pulled down both DDB1 and CUL4A, but not KEAP1 (Fig 2A and 2B), which defines NRF2 as a novel substrate of the CUL4A E3 ligase complex that operates independently of the established CUL3-KEAP1 E3 ligase machinery. We next examined the specificity of the role that WDR23 plays in the maintenance of the NRF family of transcription factors. In addition to NRF2, mammals express NRF1, NRF3, and NF-E2, which all contribute to ARE activation[51–53]. NRF1 is ubiquitously expressed, similar to NRF2, while NRF3 expression is restricted to the placenta and liver tissues, and NF-E2 is only expressed in erythrocytes. Moreover, NRF1 and NRF2 have distinct cellular roles[54, 55]. The interaction of WDR23 with NRF2 was specific, as we were unable to detect an interaction with NRF1 in cells overexpressing tagged versions of WDR23 and NRF1 (Fig 2C). As such, the WDR23-DDB1-CUL4 E3 ligase complex is specific to NRF2-dependent cytoprotection (Fig 2D).

Our experiments follow previous studies[24, 56, 57] that demonstrate that WDR23 is a component of the CUL4A-DDB1 E3 ligase complex (Fig 2A and 2B) and predict that the underlying mechanism of NRF2 regulation would be at the level of protein turnover and stability. As such, we examined whether modulating WDR23 levels could alter the abundance of NRF2 protein. The increased expression of WDR23 in HEK-293T cells decreased the abundance of co-transfected NRF2 in a dose dependent manner (Fig 2E). The reduction of NRF2 was dependent on the increase in WDR23 expression since co-transfection of a WDR23 siRNA restored NRF2 levels (S4A–S4C Fig). As predicted, the WDR23-mediated degradation of NRF2 was dependent on the ubiquitin proteasome system, as the WDR23-mediated reduction of NRF2 was attenuated when cells were treated with the proteasome inhibitor peptide MG-132 (Fig 2F). Although we are unable to detect a significant change in the rate of turnover of NRF2 when co-expressed with WDR23 (S4D Fig), this is likely complicated by the already significant reduction of NRF2 levels prior to treatment with cyclohexamide. Additionally, poly-ubiquitination signals proteins for degradation via the ubiquitin proteasome system, which led us to examine levels of ubiquinated-NRF2. To further test the functionality of WDR23 in NRF2 proteostasis, we purified the WDR23-DDB1-CUL4 complex (Fig 2A) and discovered that it could efficiently add ubiquitin chains to purified NRF2 *in vitro* (Fig 2G, S4E Fig). Taken together these data suggests that WDR23 drives NRF2 turnover by the ubiquitin proteasome system. As observed by others, we found endogenous NRF2 levels to be relatively low under basal conditions. However, overexpression of isoform 2 of WDR23 reduced endogenous NRF2 protein levels (Fig 2H). These data indicate that the regulation of NRF2 by WDR23 is in part at the level of NRF2 stability.

KEAP1 function is primarily restricted to the cytoplasm, but KEAP1-independent regulation of NRF2, perhaps in the nucleus, has long been hypothesized[58]. Between the two isoforms, there is expression of WDR23 in both the cytoplasm and nucleus, and the functional capacity of both isoforms of WDR23 suggests that its role in NRF2 regulation contributes to the unknown of KEAP1-independent mechanisms. The stable localization of WDR23 in the presence or absence of xenobiotic stress predicts that this regulation can occur regardless of the redox state of the cell. During electrophilic stress, such as treatment with oxidizing agents, the physical association of NRF2 with KEAP1 is disrupted, which stabilizes NRF2, allowing its accumulation in the nucleus[59]. We find that the interaction of WDR23 with NRF2 also occurs in cells treated with H2O2 (Fig 2I and S5A Fig), which is consistent with the idea that WDR23 regulates NRF2 independent of KEAP1. We next challenged the WDR23 regulatory system to turn over activated NRF2 following oxidative stress. In line with our studies in non-stressed cells, overexpression of WDR23 was sufficient to abrogate the increased accumulation of NRF2 following exposure to hydrogen peroxide (Fig 2J).

**Fig 3:**
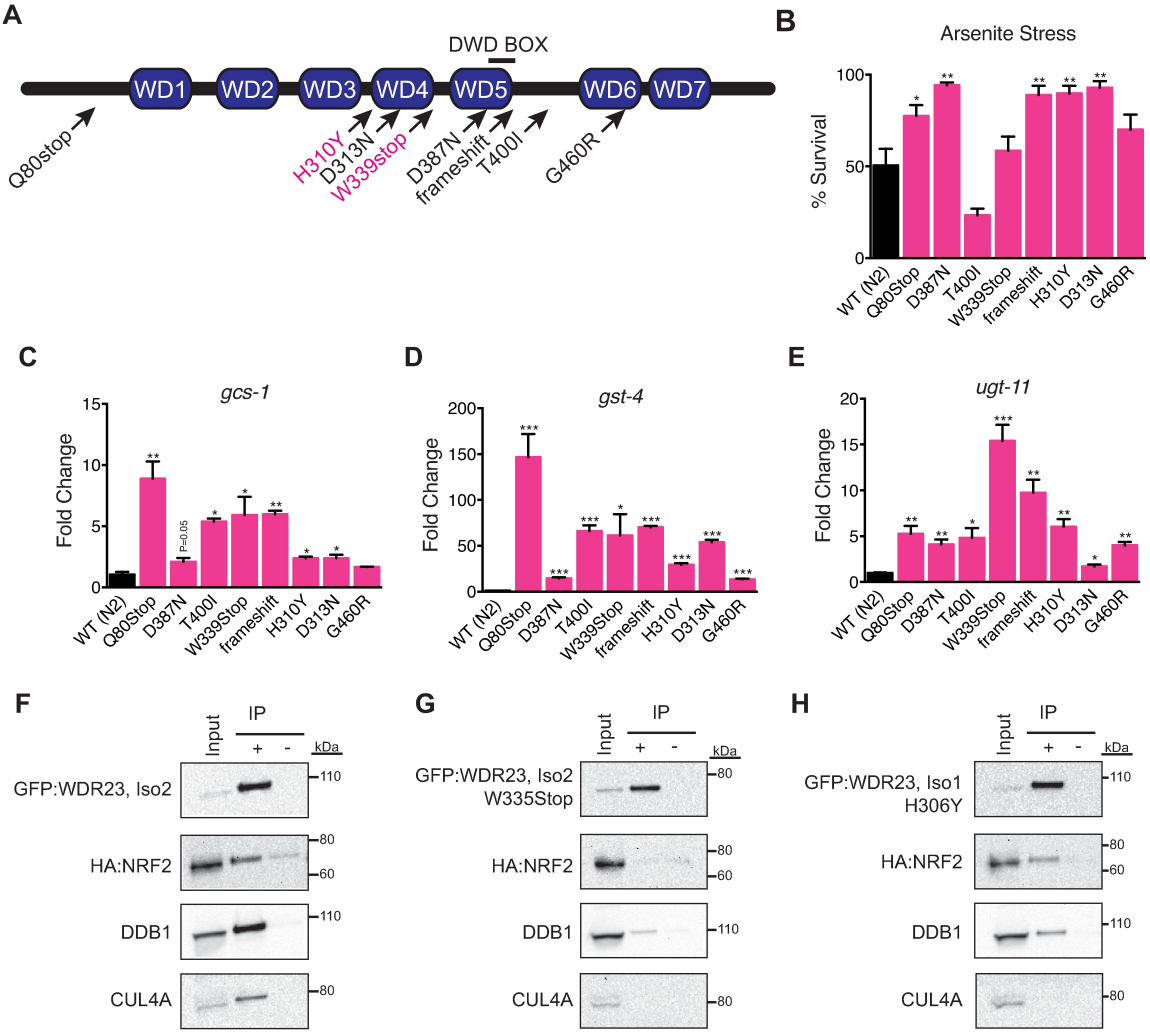
A central highly conserved domain of WDR23 facilitates binding to NRF2. (**A**) Schematic of *C*. *elegans* WDR-23 and the identity of eight recessive loss of function alleles. Conserved residues between worm and human WDR23 that are mutated for structure function analysis are in pink. (**B**) Most mutations in WDR-23 result in an increase in cytoprotection from heavy metals. WT n = 6, Q80Stop n = 5, D387N n = 6, T400I n = 6, W339Stop n = 3, frameshift n = 6, H310Y n = 6, D313N n = 6, G460R n = 6. (**C-E**) Strains harboring mutant versions of WDR-23 display increased expression of the SKN-1/NRF2 cytoprotection targets *gcs-1* (WT n = 3, Q80Stop n = 3, D387N n = 3, T400I n = 3, W339Stop n = 3, frameshift n = 3, H310Y n = 3, D313N n = 3, G460R n = 3) (**C**), *gst-4* (WT n = 3, Q80Stop n = 3, D387N n = 3, T400I n = 3, W339Stop n = 3, frameshift n = 3, H310Y n = 3, D313N n = 3, G460R n = 3) (**D**), *ugt-11* (WT n = 3, Q80Stop n = 3, D387N n = 3, T400I n = 3, W339Stop n = 3, frameshift n = 3, H310Y n = 3, D313N n = 3, G460R n = 3) (**E**). (**F-H**) As compared to cells over expressing wild type WDR23 and NRF2 (**F**), the WDR23(W335Stop) mutation (**G**) impairs binding of NRF2 and disrupts association with the DDB1-CUL4A complex, while the H306Y mutation modestly reduces binding of both (**H**). Data are mean ± s.e.m.; one-tailed *t*-test relative to control samples. *P<0.05,**P<0.01, ***P<0.001.

The conserved capacity of *C*. *elegans* WDR-23 to regulate similar cellular cytoprotection responses, albeit mediated by SKN-1, suggested we could exploit our *C*. *elegans* genetic system to identify the domains of WDR-23 that would be of functional significance for regulation of the mammalian NRF2 pathway. In *C*. *elegans*, WDR-23 is a direct regulator of SKN-1; WDR-23 delivers SKN-1 to the proteasome to regulate the abundance of the transcription factor. Therefore, we utilized the worm as a genetic tool to dissect the mechanisms behind this conserved pathway. To that end, we performed an ethyl methanesulfonate (EMS) mutagenesis screen to identify *wdr-23* mutants (S6A Fig), which we predicted would be enriched, as WDR-23 is the canonical negative regulator of SKN-1 activity in worms. We sequenced the *wdr-23* locus in all isolated mutants that mapped to linkage group I[60] and identified eight novel alleles of *wdr-23* (Fig 3A and S2 Table) that map to conserved regions of the WDR23 protein (S6B Fig)[61]. Each of these mutations is fully recessive and although variable in strength, can enhance animal survival during xenobiotic stress (Fig 3B) and activate the transcription of cytoprotection genes (Fig 3C–3E, S6C Fig) in a *skn-1*-dependent manner (S6D–S6S Fig). The mutations in *wdr-23* cluster around WD40 repeats 4 and 5, which are near the conserved DWD-box found in WDR23 across species (S3 Table). Notably, many of these mutations are in residues that are conserved from worm to man.

Our studies identify NRF2 as a substrate for the CUL4 adapter protein WDR23. Although WDR23 has previously been shown to bind to the CUL4-DDB1 complex[37, 62], the biochemical mechanism underlying this interaction is unknown. Informed by our worm mutants, we used site-directed mutagenesis to generate orthologous mutations in highly conserved residues in WDR23: H306Y and W335Stop (Fig 3A and S6B Fig). These mutant versions of WDR23 did not alter the subcellular localization of WDR23 isoform 1, but we often observed non-nuclear localized WDR23 isoform 2 harboring these mutations, which might impact their functionality (S7A–S7D Fig). Both mutations were stably expressed and could be enriched by our IP strategy, but each mutation weakened the WDR23 interaction with NRF2. The W335Stop mutation disrupted the association of both WDR23 isoforms with NRF2, DDB1 and CUL4A (Fig 3F and 3G, S7E Fig), and the H306Y mutation reduced binding to DDB1 and CUL4A and also modestly reduced NRF2 binding (Fig 3F and 3H, S7F Fig). Informed by our invertebrate studies, these results implicate a potential function of the C-terminus of WDR23 for substrate binding and recruitment of the CUL4 E3 ligase complex. Further dissection of this region will allow us to pinpoint the required residues for this interaction.

### WDR23 regulates NRF2 independently of KEAP1

**Fig 4:**
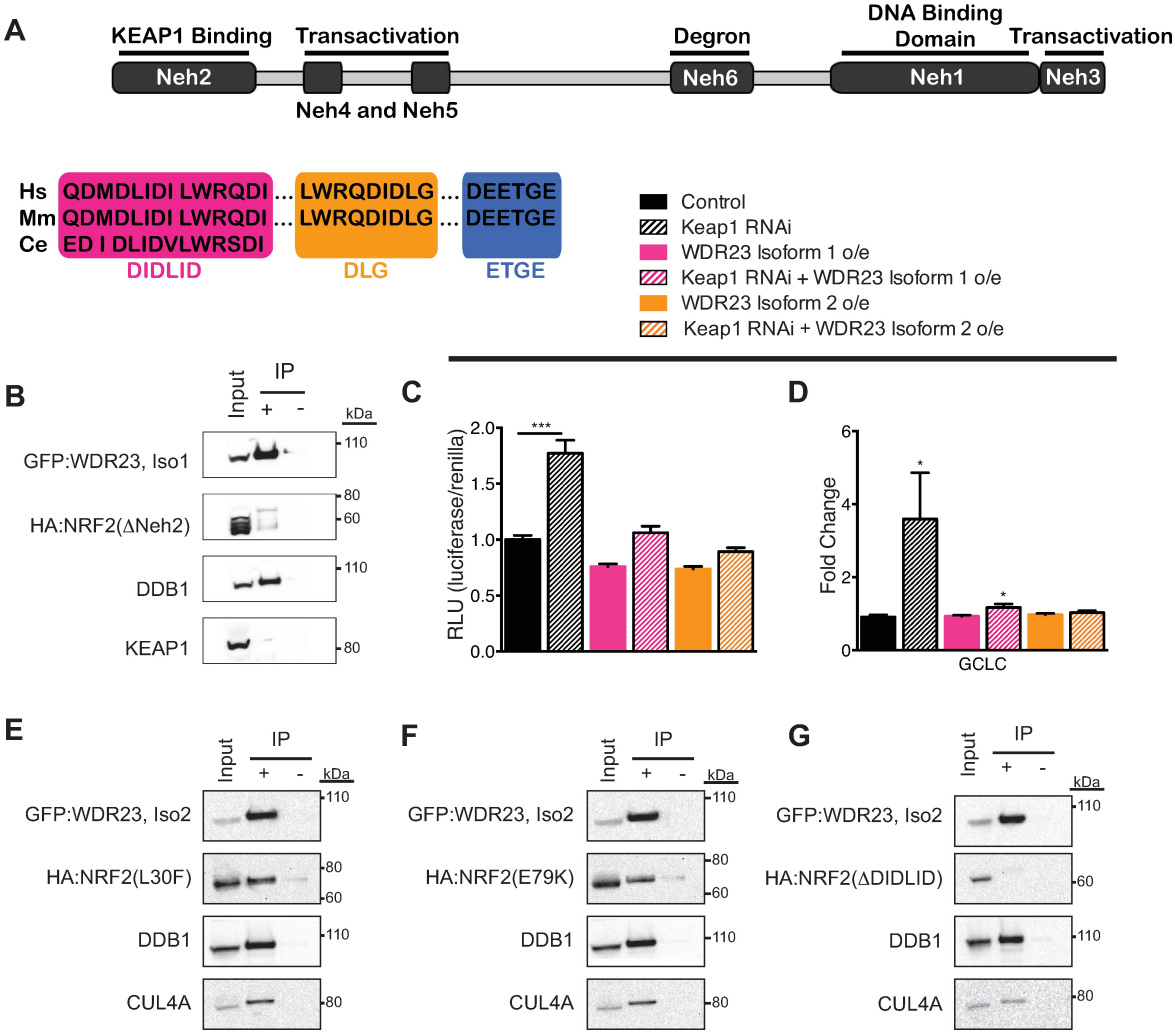
WDR23 regulates NRF2 independently of KEAP1. (**A**) Schematic of the NRF2 protein, the location of each Neh domain, and the amino acid sequence of the DIDLID, DLG, and ETGE motifs. (**B**) WDR23 requires the Neh2 domain of NRF2 for binding. Wildtype control IP in Fig 2A. (**C,D**) Reduction of *KEAP1* by RNAi induces the activation of a NRF2-dependent ARE-luciferase reporter (Control n = 32, *KEAP1* RNAi n = 32, Iso 1 n = 24, Iso 1 + Keap1 RNAi n = 24, Iso 2 n = 24, Iso 2 + *KEAP1* RNAi n = 24) (**C**) and the increased expression of the NRF2 target *GCLC* (Control n = 6, *KEAP1* RNAi n = 6, Iso 1 n = 6, Iso 1 + *KEAP1* RNAi n = 6, Iso 2 n = 6, Iso 2 + *KEAP1* RNAi n = 6) (**D**). This induction is attenuated when WDR23 is overexpressed as compared to control (GFP expression). (**E-G**) Despite shared use of the Neh2 domain by KEAP1 and WDR23 for binding, WDR23 does not require the DLG (**E**) or ETGE (**F**) motifs utilized by KEAP1, but instead requires the conserved DIDLID motif (**G**). Data are mean ± s.e.m.; one-tailed *t*-test relative to control samples. *P<0.05, ***P<0.001.

The regulation of NRF2 by KEAP1 is thought to occur in the cytoplasm[40–42]. Our immunoprecipitation studies of WDR23 did not pull down KEAP1, supporting the formation of a CUL3-KEAP1-independent regulatory complex. Six NRF2-ECH homology (Neh) domains have been defined within NRF2 that are key determinants of NRF2 regulation and activity[59, 63–66] (Fig 4A). We systematically examined a panel of NRF2 mutants, each with a different Neh domain deleted[12], and measured the capacity of WDR23 to bind the truncated protein. After transfecting tagged versions of WDR23 and the NRF2 mutants, we observed that NRF2(ΔNeh2) failed to co-IP with either WDR23 isoform (Fig 4B, S5A and S8A Figs), while binding still occurred with all other truncated versions of NRF2 (S8B–S8I Fig). This result indicates the absolute requirement of the Neh2 domain to facilitate the interaction of WDR23 with NRF2.

KEAP1 also regulates NRF2 *via* the Neh2 domain[17], which might suggest a common mechanism of WDR23 and KEAP1 regulation of NRF2, despite lack of a detectable interaction between WDR23 and KEAP1. To confirm a KEAP1-independent axis of NRF2 regulation by WDR23, we assessed the capacity of WDR23 to suppress the activation of NRF2 when KEAP1 is inhibited. Overexpression of WDR23 reduced ARE-luciferase activation in cells transfected with *KEAP1* siRNA (Fig 4C, S1 Table). Specifically, overexpression of WDR23 suppressed the induction of the canonical KEAP1-NRF2 pathway targets *GCLC* (Fig 4D), but not *NQO1* (S9A Fig).

The Neh2 domain contains 86 amino acids (Fig 4A). To better define the location where WDR23 regulates NRF2 we tested WDR23 binding to NRF2 mutants where either the first or second 43 amino acids were removed, Neh2A(Δ2–43) and Neh2B(Δ44–86). In cells overexpressing tagged version of WDR23 and Neh2 mutants, WDR23 was still able to bind Neh2B(Δ44–86), but not Neh2A(Δ2–43), indicating the binding site is the N-terminal portion of the domain (S9B–S9D Fig). This region of Neh2 contains three identifiable motifs: DIDLID, DLG, and ETGE (Fig 4A). The shared use of the Neh2 domain for binding of NRF2 by WDR23 and KEAP1 may reflect a competition between the CUL4 and CUL3 E3 ligases for NRF2 regulation. To determine whether this model was correct, we tested if WDR23 could associate with NRF2 when the motifs utilized by KEAP1 for binding were mutated[67]. Mutation of the DLG (Fig 4E and S9E Fig) or ETGE (Fig 4F, S9F Fig) motifs did not abolish binding. These findings further support the model where WDR23 can restore regulatory control of NRF2 independent of KEAP1 function (Fig 2D).

In worms, WDR-23 regulates the activity of the cytoprotective transcription factor SKN-1. SKN-1 contains a DIDLID motif that is critical for SKN-1 activity[68]. NRF2 also contains a DIDLID motif, which is found in the Neh2 domain (Fig 4A). Deletion of the DIDLID motif in NRF2 impaired WDR23 binding (Fig 4G, S9D Fig), revealing that the conserved DIDLID motif has been maintained over evolution as a mechanism of regulation. Moreover, this finding defines the DIDLID and the DLG/ETGE motifs are two independent sequences in the Neh2 domain that cooperatively regulate NRF2 by WDR23 and KEAP1, respectively.

### WDR23 impacts chemotherapy sensitivity and restores NRF2 homeostasis in cancer cells

**Fig 5:**
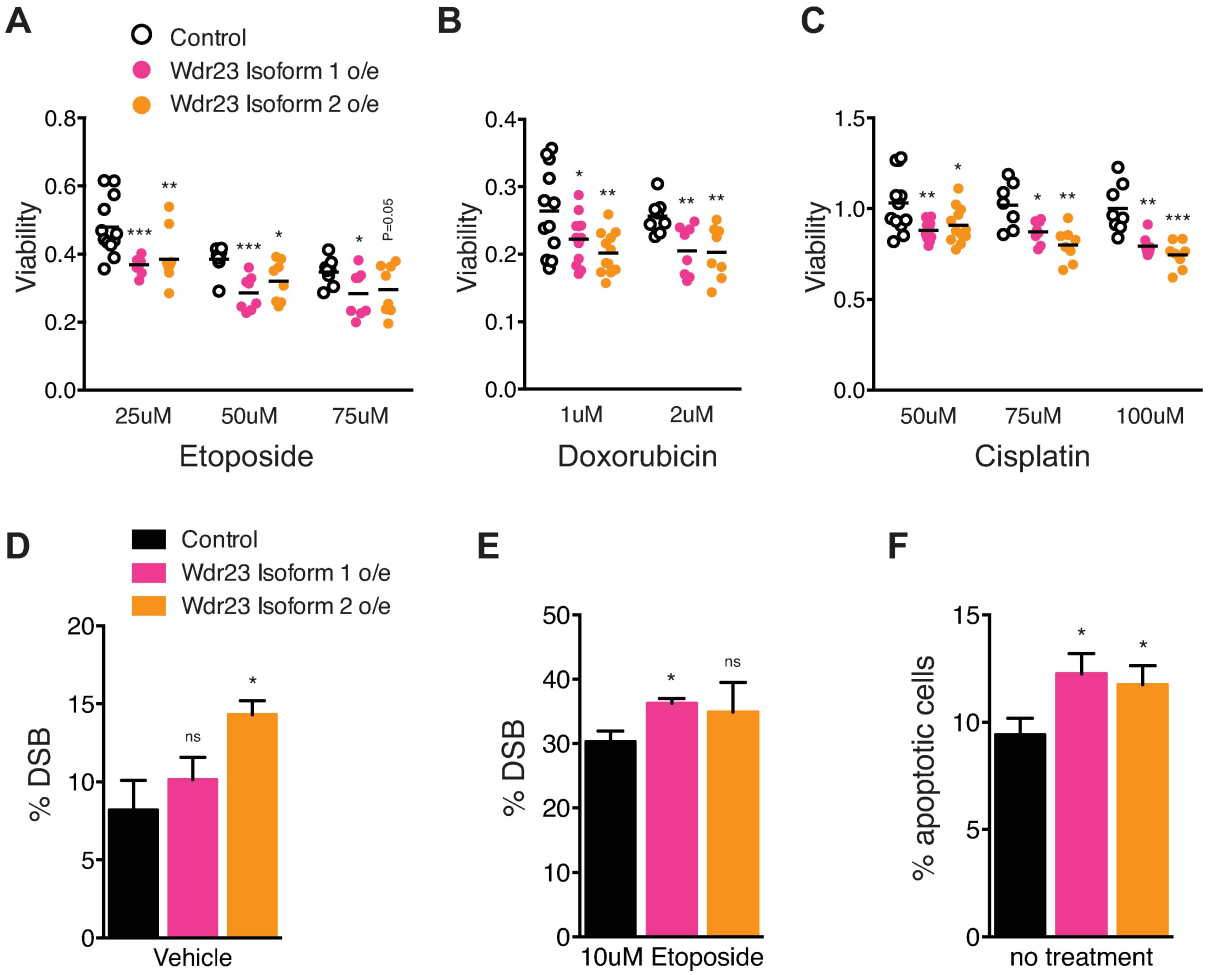
WDR23 overexpression enhances chemotherapy toxicity. (**A-C**) Cells overexpressing GFP:WDR23 isoform 1 (pink) or GFP:WDR23 isoform 2 (orange) are more sensitive to increasing concentrations of (**A**) etoposide (25uM n = 12 each, 50um n = 8 each, 75um n = 8 each), (**B**) doxorubicin (1uM n = 12 each, 2uM n = 8 each), or (**C**) cisplatin (50uM n = 12 each, 75uM n = 8 each, 100uM n = 8 each) as compared to cells expressing GFP alone (black). (**D,E**) The percentage of cells carrying DNA double strand breaks (DSB), as indicated by dual phospho-H2Ax and phospho-ATM staining, is increased when WDR23 isoform 1 or WDR23 isoform 2 is overexpressed (Control n = 3, Iso 1 n = 3, Iso 2 n = 3) and (**E**) enhances DSB incidence following etoposide treatment (Control n = 3, Iso 1 n = 3, Iso 2 n = 3). (**F**) Cellular apoptosis (Annexin V positive) is increased in cells overexpressing WDR23 isoform 1 or WDR23 isoform 2 as compared to control cells overexpressing GFP alone (Control n = 3, Iso 1 n = 3, Iso 2 n = 3). Data are mean ± s.e.m.; one-tailed *t*-test relative to control GFP overexpression for each treatment condition. *P<0.05, **P<0.01, ***P<0.001.

KEAP1 function is perturbed in several aggressive cancers that are resistant to chemo- and radiation-based therapies due to enhanced NRF2 activity, making them particularly hard to treat[69–71]. NRF2 stability leads to enhanced resistance to the cytotoxic drugs etoposide, doxorubicin, and cisplatin[72]. As such, we challenged HEK-293T cells overexpressing either GFP (control), GFP:WDR23 isoform 1, or GFP:WDR23 isoform 2 to increasing concentrations of these anti-cancer molecules. Overexpression of WDR23 isoform 1 or WDR23 isoform 2 resulted in increased sensitivity to each cytotoxic drug tested as compared to cells expressing GFP alone (Fig 5A–5C, S10A–S10D Fig and S4 Table). Cells overexpressing either isoform of WDR23 displayed a significant increase in DNA damage, as measured by dual phospho-ATM and phospho-H2Ax staining, which is indicative of DNA double strand breaks (DSB) (Fig 5D and 5E, S10E–S10J Fig). Similarly, the increased toxicity of etoposide treatment for cells overexpressing WDR23 isoform 1 was correlated with an increased DSB in those cells. Lastly, we observed enhanced apoptosis, as measured by Annexin V staining in cells overexpressing either isoform of WDR23 (Fig 5F, S10K–S10M Fig).

**Fig 6:**
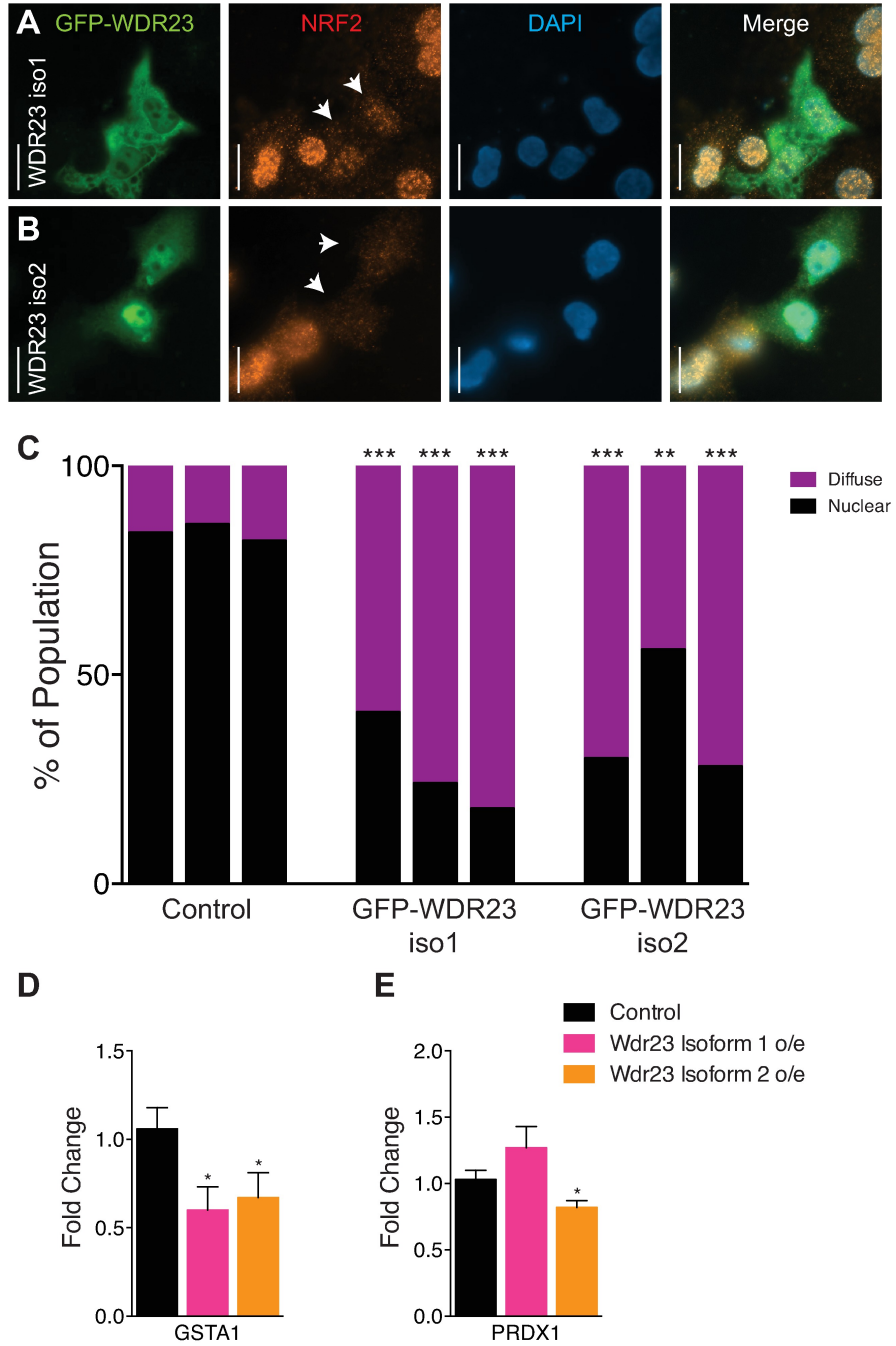
WDR23 restores NRF2 regulation in cancer cells. (**A,B**) Overexpression of GFP:WDR23 isoform 1 (**A**) or GFP:WDR23 isoform 2 (**B**) can deplete activated endogenous NRF2 (Alexa Fluor 594, red) from the nucleus (DAPI, blue) in A549 lung cancer cells. Scale bar, 20μm. Arrows denote transfected cells. (**C**) Quantification of nuclear or diffuse localization of endogenous NRF2 in cells over expressing GFP:WDR23 isoform 1 (n = 74), GFP:WDR23 isoform 2 (n = 84) or non-transfected cells from the same experiment (n = 490). Each bar represents an independent immunostaining experiment. Fisher’s exact test; **P<0.01, ***P<0.001. (**D,E**) A549 cancer cells overexpressing WDR23 have reduced levels of the NRF2 target genes *GSTA1* (Control n = 12, Iso 1 n = 6, Iso 2 n = 6) (**D**) and *PRDX1* (Control n = 12, Iso 1 n = 6, Iso 2 n = 6) (**E**). Data are mean ± s.e.m.; one-tailed *t*-test relative to control samples. *P<0.05.

**Fig 7:**
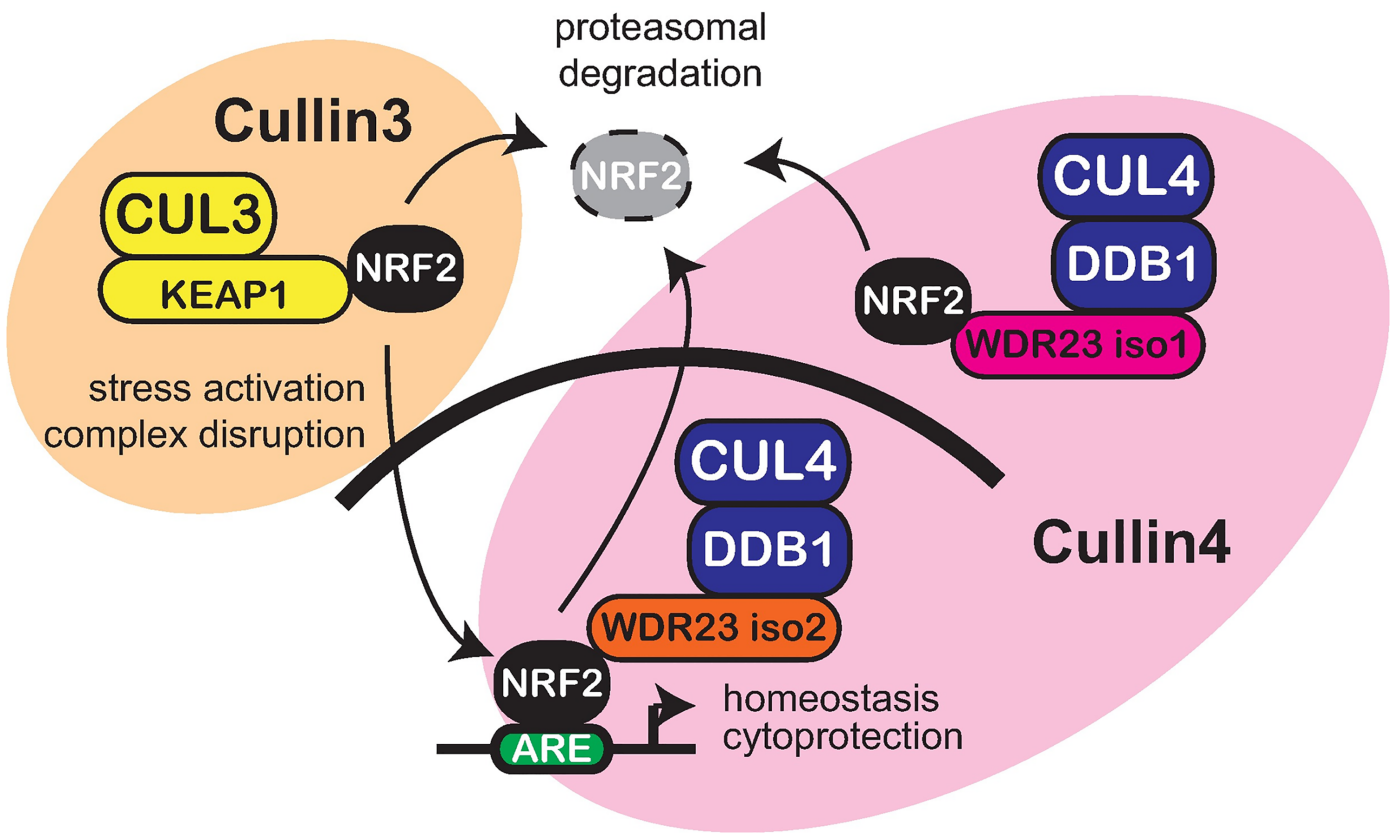
WDR23 and KEAP1 pathways coordinate regulation of NRF2. Model for the shared regulation of NRF2 by the cytoplasmic Cullin3 E3-ubiquitin ligase complex (CUL3-KEAP1) and the nuclear and the cytoplasmic Cullin4 E3-ubiquitin ligase complexes (CUL4-DDB1-WDR23).

In light of the impact WDR23 had on the NRF2 activity in untransformed cells, we predicted that WDR23 could compensate for KEAP1 loss in cancer cell lines derived from human tumors. To that end, we overexpressed either WDR23 isoform 1 or WDR23 isoform 2 in A549 lung carcinoma cells, where loss of KEAP1 results in NRF2 nuclear accumulation. We exploited the transient transfection system, which advantageously facilitated a side-by-side comparison of cell expressing WDR23 to those without. In support of our hypothesis, cells transfected with either isoform of WDR23 had reduced endogenous nuclear NRF2, while NRF2 in non-transfected cells remained nuclear (Fig 6A–6C). Moreover, when looking at the immunostaining of individual cells, the reduction of nuclear NRF2 was more pronounced when WDR23 isoform 2 was overexpressed, and is consistent with the idea that the WDR23 is the nuclear complement to the cytoplasmic KEAP1 system. Additionally, we also examined the effect of WDR23 overexpression in H460 human non-small-cell lung carcinoma cells, which also harbor a KEAP1 mutation (different from that of A549) that results in nuclear NRF2[73]. H460 cells that overexpress either isoform of WDR23 also had reduced endogenous nuclear NRF2, (S11F and S11G Fig), in line with what we observed in A549 cells. Quantification of total NRF2 protein levels in A549 cells overexpressing WDR23 isoform 1 or WDR23 isoform 2 reveal a reduction of approximately 20%, although based on the difficulty in transfection of these cells, and the resulting mosaic nature of the population, this is likely an underestimate of the effect WDR23 has on NRF2 stability (S11D and S11E Fig). Lastly, A549 lung carcinoma cells that overexpress either isoform of WDR23 have reduced expression of the NRF2 targets GSTA (Fig 6D) and PRDX1 (Fig 6E), which are often induced in cancer cells and have been identified as potential targets for directed therapy[74, 75]. Collectively, our studies provide new mechanistic insight underlying the complex regulation of NRF2-dependent cytoprotection (Fig 7). Additionally, these findings are of particular medical relevance as the ability to shutdown NRF2 activity, independently from KEAP1, is of particular clinical interest for cancers where activated NRF2 contributes to both the severity and resistance to treatment by radiation- or chemo-based therapies.

## Discussion

Exposures from multiple sources—both environmental and internal—impact a person’s overall health and susceptibility to disease, with the total exposure throughout an individual’s lifespan (conception to death) defined as the exposome. The mechanisms underlying responses to the exposome are central to our understanding of human health and disease. Collectively, cellular cytoprotective systems, including NRF2, are required for appropriate responses to the exposome. However, these response systems require precise regulation, both for activation and inactivation; inappropriate activation of these pathways can also promote resistance to the inherently toxic treatment of diseases by chemo- and radiation therapies.

We propose a new regulatory system to maintain NRF2-dependent cellular homeostasis. Cellular adaptation to stress (oxidative, xenobiotic, dietary) is essential to ensure organismal survival, and NRF2 is an exceptionally well-studied and key determinant of cellular stress responses[2, 11]. Our findings expand upon 15 years of research that have focused primarily on the role of the KEAP1-CUL3 E3-ubiquitin ligase proteasome system as the preeminent mechanism for negative regulation of NRF2[10, 76, 77]. Through the combined use of *C*. *elegans* and human cell culture models, we establish a functional and evolutionarily conserved role for human WDR23 that can regulate NRF2 levels and which operates independently of the canonical KEAP1-CUL3 pathway.

Gene dosage is a well-documented genetic tool and physiologically is of critical importance for cancer cell biology[78]. Our combined use of both gene overexpression, RNAi knockdown, and genetic ablation of WDR23 collectively reveal a previously unknown axis of regulation for NRF2 protein levels. Based on the ability of WDR23 to regulate NRF2, we predicted that mutations in WDR23 could be important for cancer cell biology. To that end, we queried the Catalogue Of Somatic Mutations In Cancer (COSMIC) online database of somatically acquired mutations found in human tumor samples for evidence of deregulated WDR23[79, 80]. 103 unique somatic mutations in WDR23 have been documented that include 9 nonsense and 74 missense mutations discovered across multiple tissues (S5 Table). Several of these mutations fall within the region of WDR23 that we have defined as important for substrate binding and association with DDB1 (S11A Fig). In addition, WDR23 expression is increased in 427 tumor samples, including 40 with increased copy number, and decreased in 279 samples, including 9 with reduced copy number (S5 Table). In support of our finding that WDR23 negatively regulates NRF2, several of these cancer cells with mutations in WDR23 have increased expression of NRF2 targets, but have normal KEAP1. The impact that these WDR23 mutations and variation in expression play in cancer cell physiology will be of great interest.

Our data, when combined with the information archived at the COSMIC from human somatic tumors, strongly supports the prediction that enhancing the WDR23 pathway could reestablish regulation of activated NRF2 in KEAP1(-/-) cancer cells. It would be of interest to determine the extent by which WDR23 can impact homeostasis in both normal and transformed cells, with and without functional KEAP1. Cancers with loss of KEAP1 pose a serious complication for clinical treatment due to the NRF2-related etiology of the resistance to classical treatments. We find that the additional expression of WDR23 is sufficient to enhance cellular sensitivity to three prescribed cytotoxic anticancer drugs: etoposide, doxorubicin, and cisplatin. The sensitivity to these drugs is correlated to increased DNA DSB (Fig 5D and 5E) and cellular apoptosis (Fig 5F). Future translational studies to combine chemotherapy with WDR23 negative regulation of NRF2 will revolutionize the treatment of cancers with enhanced cytoprotection.

It is well established that NRF2 regulates the expression of a diverse collection of targets but the mechanism of selection of one ARE-containing target over another remains unknown[27, 44–47]. In a simple two-component model of regulation (KEAP1 and NRF2), this was difficult to reconcile, however, our discovery of WDR23 as a second layer of regulation could explain the differential regulation of certain NRF2 targets over others. Our findings support a model where WDR23 and KEAP1 can each regulate NRF2 levels by independent mechanisms. Feedback regulation of redundant or parallel pathways can occur at the level of transcription when one arm of the system is disabled[81–83] or when demand on the pathway is increased, as observed in our transcriptional analysis of increased KEAP1 during oxidative stress when WDR23 is overexpressed in mammalian cells (S2I Fig)[6, 84] and the increased expression of *wdr-23* in *C*. *elegans wdr-23* mutants (S6B Fig). As such, we next investigated whether WDR23 expression is altered in tumor samples harboring KEAP1 mutations and *vice versa*. In support of our hypothesis, several lung cancers harboring KEAP1 mutations have increased expression of WDR23, ranging from 2.06 to 4.8-fold (S11B Fig). Similarly, multiple stomach cancer samples that have sequence identified somatic mutations in WDR23 have increased KEAP1 expression, ranging from 2.07 to 3.41 fold (S11C Fig). Further delineation of the cross talk and specificity of the WDR23-CUL4 and KEAP1-CUL3 pathways will be of critical importance.

The evolutionary maintenance of the WDR23 pathway from invertebrates to humans and the absence of KEAP1 in the *C*. *elegans* genome suggest that the multilayer regulation of NRF2 in mammals evolved in parallel to organism complexity. The existence of independent mechanisms to control NRF2 activity is intriguing from an evolutionary and molecular biology perspective. Although the necessity for two parallel pathways remains unknown, their existence might be required to enhance the magnitude of the stress response, refine the type of stress response induced, or control tissue specific roles.

The diversity of cellular and organismal functions that are influenced by NRF2 activity demands a more thorough understanding of complexities underlying NRF2 regulation. Our findings have enhanced our appreciation of the complex nature of NRF2-dependent stress adaptation and will lay the foundation for the development of new therapeutics to appropriately tailor a person’s exposome responses.

## Materials and methods

### Cell cultures, transfections, and chemicals

Cell cultures were maintained as previously described[85]. HEK-293T and HepG2 cells were maintained in Dulbecco’s modified Eagle’s medium supplemented with 10% fetal bovine serum and 1% antibiotic/antimycotic (Thermo Fisher) at 37°C, 5% CO2. A549 (ATCC) cells were maintained in Ham’s F-12K (Kaighn’s) medium supplemented with 10% fetal bovine serum and 1% antibiotic/antimycotic at 37°C, 5% CO2. H460 (ATCC) cells were maintained in RMPI-1640 medium supplemented with 10% fetal bovine serum and 1% antibiotic/antimycotic at 37°C, 5% CO2. Transfections were performed with Lipofectamine 3000 (Thermo Fisher) according to the manufacturer’s protocol. siRNAs (Thermo Fisher) used include: KEAP1 (HSS114799, HSS114800, HSS190639), WDR23 (HSS129631, HSS129632, HSS129633), NRF2 (s9492). For genes with more than one siRNA listed, a cocktail mixture of the previously mentioned siRNAs is used for efficient knockdown. Chemical treatments include: 50μM tert-Butylhydroquinone (Sigma) and 250μM H2O2 (Sigma). 50μM tert-Butylhydroquinone (Sigma), 250μM H2O2 (Sigma), and 10μM MG-132 (Sigma).

### Recombinant DNA

Full-length cDNA sequence of *Hs* Wdr23 Isoforms 1 and 2 and *Ce wdr-23* Isoforms A and B were cloned into pcDNA 6.2/N-EmGFP/TOPO (Thermo Fisher). 3xFLAG:Nrf2 and 3xFLAG:Nrf1 were purchased from GeneCopeia. mCherry-LaminA-C-18 was a gift from Michael Davidson (Addgene plasmid # 55068). Nrf2(ΔNeh) plasmids were a generous gift from Donna Zhang (University of Arizona). Additional mutants were generated from existing plasmids using Q5 Site-Directed Mutagenesis (NEB).

### *C*. *elegans* strains utilized and culture methods

*C*. *elegans* were cultured using standard techniques[86]. The following strains were used: wild-type N2 Bristol, CL2166[*gst-4p*::*gfp*], SPC296[*wdr-23(lax101;Q80Stop)*], SPC318[*wdr-23(lax123;D387N)*], SPC302[*wdr-23(lax124;T400I)*], SPC306[*wdr-23(lax126;W339Stop)*], SPC299[*wdr-23(lax129;frameshift)*], SPC315[*wdr-23(lax134;H310Y)*], SPC303[*wdr-23(lax211;D313N)*], and SPC317[*wdr-23(lax213;G460R)*]. Double mutants were generated by standard genetic techniques. For RNAi experiments, NGM plates containing 5 mM IPTG and 100 μg ml^-1^ carbencillin were seeded with overnight cultures of double-stranded RNAi-expressing HT115 bacteria. Plates were allowed to induce overnight followed by transfer of age-synchronous populations of *C*. *elegans*. For arsenite survival, L4 worms of indicated genotype were transferred to plates containing 5mM arsenite (J.T.Baker) and counted for survival after 24 hours.

### Isolation of *wdr-23* mutants

Ethyl methanesulfonate (EMS) mutagenesis was performed as previously described[25]. Briefly, a *C*. *elegans* strain harboring the SKN-1 transcriptional reporter *gst-4p*::*gfp* was mutagenized with EMS, and F1 worms with high GFP expression (indicating SKN-1 activation) were selected. A complementation group of eight recessive alleles were isolated and mapped to chromosome I. The *wdr-23* gene was sequenced in each mutant isolated to determine the specific mutation in each strain.

### Fluorescent imaging

Cells were grown on coverslips coated with poly-D-lysine (Corning) and transiently transfected with indicated plasmids. Twenty-four hours post-transfection, coverslips were mounted on cover slides and imaged with a Zeiss Axio Imager.M2m microscope, Axio Cam MRm camera, and Zen Blue software.

### RNA extraction and quantitative PCR

Quantitative PCR was performed as previously described[27]. Briefly, either human cells or worms of the indicated genotypes and treatments were collected and lysed in Tri reagent (Zymo Research). RNA was extracted according to the manufacturer’s protocol. DNA contamination was digested with DNase I and subsequently, RNA was reverse-transcribed to complementary DNA using qScript cDNA SuperMix (Quanta Biosciences). Quantitative PCR was performed by using SYBR Green (BioRad). The expression levels of *​snb-1* and B2M were used to normalize samples in worms and human cells, respectively. Primer sequences are listed in Supplemental S6 Table and raw data in S7 Table.

### Luciferase reporter gene assay

HEK-293T cells were transiently transfected with the indicated plasmids and/or siRNA and Cignal antioxidant response luciferase reporter (Qiagen). Forty-eight hours post-transfection, cells were assayed using the Dual-Glo Luciferase Assay System (Promega) according to the manufacturer’s protocol. Firefly luciferase activity was normalized to renilla luciferase activity (S8 Table).

### Co-immunoprecipitation

HEK-293T cells were transiently transfected with indicated plasmids. Twenty-four hours post-transfection and without treatment of the proteasome inhibitor MG-132, cells were lysed in 0.5% CHAPS buffer (10mM Tris/Cl pH 7.5, 150mM NaCl, 0.5mM EDTA, 0.5% CHAPS) containing Halt Protease Inhibitor (Thermo Fisher). Immunoprecipitation of GFP:WDR23 was performed according to the manufacturer’s protocol (ChromoTek). Briefly, cell lysates were precleared with blocked magnetic agarose GFP Trap beads for 1 hour at 4°C, followed by incubation with magnetic agarose GFP Trap beads for 1 hour at 4°C. After three washes (1mM Tris/Cl pH 7.5, 150mM NaCl, 0.5mM EDTA) post-immunoprecipitation, immunoprecipitated protein complexes were eluted in 2X sample buffer (0.1M Tris/Cl pH 6.8, 4% SDS, 20% glycerol, 0.2M DTT, 0.1% bromophenol blue) by boiling for 10 minutes at 95°C. Samples were analyzed by Western blot.

### Western blot analysis and antibodies

For detection of protein expression in total cell lysates, cells were lysed in RIPA buffer (50mM Tris/Cl pH 8, 150mM NaCl, 0.5% sodium deoxycholate, 0.1% SDS with Halt Protease inhibitor (Thermo Fisher)). Protein concentrations were measured with Bradford (Amaresco), then prepared with 5X sample buffer (0.25M Tris/Cl pH 6.8, 10% SDS, 50% glycerol, 0.5M DTT, 0.25% bromophenol blue), electrophoresed through Bolt 4–12% bis-tris polyacrylamide gels in MOPS running buffer (Thermo Fisher), transferred to nitrocellulose membranes, and subjected to immunoblot analysis. Antibodies used include: GFP GF28R (Thermo Fisher), FLAG M2 (Sigma), NRF2 H-300 (Santa Cruz), NRF2 C-20 (Santa Cruz), DDB1 A300-462 (Bethyl), CUL4A 113876 (GeneTex), KEAP1 ab66620 (Abcam), Actin A5441 (Sigma), Tubulin 21485 (CST), Ubiquitin 1859660 (Thermo Fisher).

### Immunocytochemistry

A549 cells were grown on coverslips coated with poly-D-lysine (Corning) and transiently transfected with indicated plasmids. Forty-eight hours post-transfection, cells were fixed in 100% methanol in -20°C for 5 minutes, blocked in 10% normal goat serum/PBS for 20 minutes, incubated in primary antibody for 1 hour each, incubated in secondary Alexa Fluor antibody (Abcam) for 1 hour each, and mounted with Vectashield with DAPI (Vector Labs). Images were taken with a Zeiss Axio Imager.M2m microscope, Axio Cam MRm camera, and Zen Blue software (S9 Table).

### Chemotherapy treatment and cytology

HEK-293T cells were transiently transfected with indicated plasmids. Forty-eight hours post-transfection, cells were treated with the indicated chemical: Etoposide (Cayman), Doxorubicin (Cayman), or Cisplatin (Cayman). For viability assays, after forty-eight hours of treatment, cells were assayed using the Vybrant MTT Cell Proliferation Assay Kit (Thermo Scientific), performed according to the manufacturer’s protocol. Viability was calculated relative to the vehicle treatment for each transfection condition. For DNA damage analysis, after twenty-four hours of treatment, cells were assayed using the Muse Multi-Color DNA Damage Kit (EMD Millipore) and Muse Cell Analyzer (EMD Millipore), performed according to the manufacturer’s protocol. For apoptotic cell analysis, cells were assayed using the Muse Annexin V and Dead Cell Assay Kit (EMD Millipore) and Muse Cell Analyzer (EMD Millipore), performed according to the manufacturer’s protocol.

### *In vitro* ubiquitylation assay

Reactions were performed as described in Broderson M. M. L. et al. (2016), with the exception of NRF2 eluate from a PURExpress *In Vitro* Protein Synthesis Kit (NEB). Briefly, Flag tagged NRF2 was purified from HEK-293T cells with Flag-M2 affinity resin (Sigma) for Fig 2G or 250 ng of HALO-NRF2 was incubated with PURExpress components in a 25 μl reaction for 3 hours at 37°C for S4E Fig and 5 μl was used for each in vitro ubiquitylation reaction. CUL4 ligase (DDB1, CUL4A, WDR23) purification is described above.

### Statistics

Statistical analyses were performed with GraphPad Prism 6 software. Data are presented as mean ± s.e.m. Data were analyzed by using unpaired Student’s t-test, one-way ANOVA, and Fisher’s exact test, where indicated. P<0.05 was considered as significant.

## Supporting information

S1 Fig WDR23 is a conserved protein. (**A**) Homology table of selected WDR23 proteins with BLAST e-values among invertebrates and vertebrates. (**B-E**) Subcellular localization of human WDR23 isoform 1 in the cytoplasm and nucleus (**B**) and WDR23 isoform 2 primarily in the nucleus (**C**) in HepG2 cells overexpressing GFP-tagged WDR23 is similar to the overexpression of these same constructs observed in HEK-293T cells (Fig 1) and of worm WDR-23A (**D**) and WDR-23B (**E**), respectively. (**F-G**) The subcellular localization of overexpressed GFP:WDR23 isoform 1 (**F**) and GFP:WDR23 isoform 2 (**H**) in untreated cells is not measurably altered in cells treated with tBHQ and overexpressing GFP:WDR23 isoform 1 (**G**) or GFP:WDR23 isoform 2 (**I**).
(PDF)

S2 Fig Specificity of impact of WDR23 of NRF2 cytoprotection. (**A,B**) Overexpression of *C*. *elegans* WDR-23A or WDR23B is unable to alter NRF2 transcriptional responses in normal cells (Control n = 16, Ce WDR-23A n = 16, Ce WDR-23B n = 16) (**A**) or in cells with reduced *KEAP1* expression following *KEAP1* siRNA treatment (Control n = 16, *KEAP1* RNAi n = 16, Ce WDR-23A n = 16, *KEAP1* RNAi + Ce WDR-23A n = 16, Ce WDR-23B n = 16, *KEAP1* RNAi + Ce WDR-23B n = 15) (**B**). (**C-E**) Overexpression of WDR23 isoform 1 or isoform 2 does not significantly reduce the expression of the NRF2 targets *GCLM* (Control n = 9, Iso 1 n = 6, Iso 2 n = 3) (**C**), *ABCC1* (Control n = 9, Iso 1 n = 6, Iso 2 n = 3) (**D**) or *CYP4A11* (Control n = 9, Iso 1 n = 6, Iso 2 n = 3) (**E**) in the absence of stress. (**F-H**) The increased expression of NRF2 targets following exposure to tBHQ does not occur for *PRDX1* (Control n = 9, +tBHQ n = 9, Iso 1 n = 6, Iso 1 +tBHQ n = 6, Iso 2 n = 3, Iso 2 +tBHQ n = 3) (**F**) when WDR23 is ectopically expressed while expression of *NQO1* (Control n = 9, +tBHQ n = 9, Iso 1 n = 6, Iso 1 +tBHQ n = 6, Iso 2 n = 3, Iso 2 +tBHQ n = 3) (**G**) and *HO-1* (Control n = 9, +tBHQ n = 9, Iso 1 n = 6, Iso 1 +tBHQ n = 6, Iso 2 n = 3, Iso 2 +tBHQ n = 3) (**H**) is still increased. (**I**) The compensatory increased expression of *KEAP1* following stress is attenuated when WDR23 isoform 1 or isoform 2 are overexpressed. (Control n = 9, +tBHQ n = 9, Iso 1 n = 6, Iso 1 +tBHQ n = 6, Iso 2 n = 3, Iso 2 +tBHQ n = 3) (**J**) *Wdr23* knockout (KO) MEF cells display increased expression of *Keap1* (Control n = 5, KO n = 12). (**K,L**) *Slbp* siRNA treatment does not significantly alter transcript levels of (**M**) NRF2 or the NRF2 transcriptional targets (**N**) *Nqo1*, (**O**) *Ho-1*, (**P**) *Gclc*, or (**Q**) *Acadl* in HEK293T cells. (Control n = 4, *Slbp* RNAi n = 3).
(PDF)

S3 Fig Control experiments performed for co-IP studies shown in main figures. (**A**) Overexpression of GFP and subsequent IP of the GFP protein does not pull down NRF2, DDB1, CUL4A, or KEAP1. (**B**) Schematic NRF2 protein and localization of the binding site of a NRF2 siRNA and domains used as antigens H-300 and C-20 for the production of NRF2 specific antibodies. (**C,D**) Specificity of the NRF2 protein detected in co-IPs when detected by H-300 (**C**) and C-20 (**D**).
(PDF)

S4 Fig WDR23 expression reduces NRF2. (**A**-**C**) The decreased level of NRF2 protein when WDR23 is expressed is dependent on WDR23 and reversed by WDR23 siRNA treatment (**A**), which efficiently reduces WDR23 mRNA levels (**B**), and also reduces endogenous WDR23 protein (**C**). (**D**) NRF2 protein levels are reduced when WDR23 is expressed; however a significant change in turnover rate is not detected when cells are treated with cyclohexamide (CHX). (**E**) Time dependent polyubiquitination of NRF2 by the CUL4-DDB1-WDR23 complex. *, non-specific cross reacting band.
(PDF)

S5 Fig Persistence of WDR23-dependent regulation of NRF2 during stress. (**A**) The interaction of WDR23 isoform 1 or WDR23 isoform 2 with NRF2 occurs even in the presence of oxidative stress.
(PDF)

S6 Fig Identification of conserved domains in WDR23 by *C*. *elegans* genetic screens. (**A**) Schematic of EMS mutagenesis screen to identify *wdr-23* mutations, which lead to activation of the SKN-1 reporter *gst-4*::*gfp*. (**B**) Location of conserved mutations in the crystal structure of DCAF provided by the Protein Model Portal[61]. (**C**) Strains harboring mutant versions of WDR-23 display compensatory increased expression of *wdr-23* itself. (WT n = 3, Q80Stop n = 3, D387N n = 3, T400I n = 3, W339Stop n = 3, frameshift n = 3, H310Y n = 3, D313N n = 3, G460R n = 3). (**D-S**) The increased expression of the SKN-1/NRF2 transcriptional reporter *gst-4*::*gfp* in *wdr-23* mutants (**D,F,H,J,L,N,P,R**) is dependent on *skn-1* (**E,G,I,K,M,O,Q,S**).
(PDF)

S7 Fig Mutations in WDR-23 alter subcellular localization. (**A-D**) The H306Y (**A**,**B**) and W335Stop (**C,D**) mutations do not measurably change the subcellular localization of WDR23 isoform 1 while the same mutations in WDR23 isoform 2 leads to more cytoplasmic protein. (**E**) The W335Stop mutation in WDR23 isoform 1 reduces the interaction with NRF2 and DDB1-CUL4 complexes. (**F**) The H306Y mutation in WDR23 isoform 2 modestly reduces the interaction with NRF2 and DDB1-CUL4.
(PDF)

S8 Fig The interaction of WDR23 with NRF2 is dependent on the Neh2 domain. (**A**) WDR23 isoform 2 does not interact with NRF2ΔNeh2. (**B-I**) WDR23 isoform 1 (**B,D,F,H**) or WDR23 isoform 2 (**C,E,G,I**) still interact with NRF2ΔNeh4,5 (**B,C**), NRF2ΔNeh6 (**D,E**), NRF2ΔNeh1 (**F,G**), and NRF2ΔNeh3 (**H,I**).
(PDF)

S9 Fig WDR23 binding of NRF2 is independent of KEAP1. (**A**) Although overexpression of WDR23 can suppress some NRF2 transcriptional targets when *KEAP1* is reduced, *NQO1* expression remains high. (Control n = 6, *KEAP1* RNAi n = 6, Iso 1 n = 6, Iso 1 + *KEAP1* n = 6, Iso 2 n = 6, Iso 2 + *KEAP1* RNAi n = 6). (**B-C**) Deletion of the first half of the Neh2 domain (ΔNeh2A) abolishes binding to WDR23 (**B**), while deletion of the second half of the Neh2 domain (ΔNeh2B) can still interact (**C**). (**D**) Single blot assessment of differential binding of WDR23 to full length NRF2, ΔNeh2A, and ΔDIDLID. (**E,F**) Mutation of the DLG (**E**) or ETGE (**F**) motifs in the Neh2 domain of NRF2 does not abolish binding by WDR23.
(PDF)

S10 Fig WDR23 enhances cellular sensitivity to cytotoxicity. (**A**) Viability of cells with treatment of the vehicle DMSO. (Control n = 18, Iso 1 n = 20, Iso 2 n = 20) (**B-D**) Cells overexpressing GFP:WDR23 isoform 1 (pink) or GFP:WDR23 isoform 2 (orange) are more sensitive to etoposide (100uM n = 12 each, 150uM n = 8 each) (**B**), doxorubicin (0.25uM n = 12 each, 0.5uM n = 12 each, 0.75uM n = 4 each) (**C**), and cisplatin (Control n = 10, Iso 1 n = 12, Iso 2 n = 12) (**D**). (**E-J**) Cells overexpressing GFP:WDR23 isoform 1 (**F**) or GFP:WDR23 isoform 2 (**G**) have increased overall levels of DNA double-stranded breaks compared to cells overexpressing GFP alone (**E**), and upon treatment with etoposide, cells overexpressing GFP:WDR23 isoform 1 (**I**) or GFP:WDR23 isoform 2 (**J**) results in enhanced DNA double-stranded breaks compared to cells overexpressing GFP alone (**H**). (**K-M**) Cells overexpressing GFP:WDR23 isoform 1 (**L**) or GFP:WDR23 isoform 2 (**M**) have increased number of apoptotic cells compared to cells overexpressing GFP alone (**K**). Data are mean ± s.e.m.; one-tailed t-test relative to control GFP overexpression for each treatment condition.
(PDF)

S11 Fig Deregulation of WDR23 in human somatic tumors. (**A**) Location of WDR23 mutations sequence-confirmed from somatic tumors in human patients. Pink lines are in regions identified in *C*. *elegans* and human in this study that result in SKN-1/NRF2 activation. (**B**) Somatic tumors isolated from lung cancer patients with confirmed KEAP1 mutations have increased expression of WDR23. (**C**) Samples from stomach cancers with confirmed WDR23 mutations have increased expression of KEAP1. (**D,E**) Total NRF2 protein is reduced in A549 cells transiently transfected with WDR23 isoform 1 or isoform 2; quantified in (**E**). (**F**) Increased expression of GFP:WDR23 isoform 1 or isoform 2 can deplete activated endogenous NRF2 (Alexa Fluor 594, red) from the nucleus (DAPI, blue) in H460 cells. Scale bar, 20um. (**G**) Quantification of nuclear or diffuse localization of endogenous NRF2 in H460 cells overexpressing GFP:WDR23 isoform 1 (n = 188), GFP:WDR23 isoform 2 (n = 130), or GFP (n = 225) from the same experiment shown. Each bar represents an independent immunostaining experiment. Fisher’s exact test; **P<0.01, ***P<0.001.
(PDF)

S1 Table RNAi efficiencies. (PDF)

S2 Table *C*. *elegans* wdr-23 mutants. (PDF)

S3 Table DWD-box motif homology. (PDF)

S4 Table Raw MTT cell survival assay data. (PDF)

S5 Table COSMIC database analysis of WDR23 mutations and expression in somatic tumors. (PDF)

S6 Table qPCR primer sequences. (PDF)

S7 Table qPCR values. (PDF)

S8 Table Luciferase assay values. (PDF)

S9 Table Immunostaining values. (PDF)
