# Supplementary material for: WDR23 regulates NRF2 independently of KEAP1
Source: PLoS Genet. 2017 Apr 28;13(4):e1006762. doi: 10.1371/journal.pgen.1006762 (PMC5428976; doi:10.1371/journal.pgen.1006762)
Supplement: S1 Fig — (A) Homology table of selected WDR23 proteins with BLAST e-values among invertebrates and vertebrates. (B-E) Subcellular localization of human WDR23 isoform 1 in the cytoplasm and nucleus (B) and WDR23 isoform 2 primarily in the nucleus (C) in HepG2 cells overexpressing GFP-tagged WDR23 is similar to the overexpression of these same constructs observed in HEK-293T cells (Fig 1) and of worm WDR-23A (D) and WDR-23B (E), respectively. (F-G) The subcellular localization of overexpressed GFP:WDR23 isoform 1 (F) and GFP:WDR23 isoform 2 (H) in untreated cells is not measurably altered in cells treated with tBHQ and overexpressing GFP:WDR23 isoform 1 (G) or GFP:WDR23 isoform 2 (I). (PDF) [file pgen.1006762.s001.pdf]

A

BLAST e-value	Species	Hit	Description	% Length
**Invertebrates**				
3.4e-224	*C. brenneri*	CN:CN26609	CBN17984	90.4%
1.9e-217	*C. briggsae*	BP:CBP37043	CBR-WDR-23	93.3%
4.1e-86	*P. pacificus*	PP:PP43443	PPA-WDR-23	65.5%
7.8e-86	*B. malayi*	BM:BM31075	Bm3045, isoform e	73.0%
1.7e-82	*O. volvulus*	OV:OVP07517	OVOC1095	71.6%
1.4e-79	*C. japonica*	JA:JA42848	CJA27743	46.1%
9.1e-75	*C. remanei*	RP:RP41093	CRE-WDR-23	37.0%
1.1e-13	*D. melanogaster*	FLYBASE:CG9945	Flybase gene name is CG9945-PA	78.6%
4e-09	*S. cerevisiae*	SGD:YCR072C	WD-repeat protein involved in ribosome biogenesis; may interact with ribosomes; required for maturation and efficient intra-nuclear transport or pre-60S ribosomal subunits, localizes to the nucleolus	26.4%
**Vertebrates**				
9.8e-83	*H. sapiens*	ENSEMBL:ENSP00000380146	Isoform 2 of DDB1- and CUL4-associated factor 11	65.1%
4.9e-82	*R. norvegicus*	SW:Q5M9G8	DDB1- and CUL4-associated factor 11	65.1%
4.9e-82	*B. taurus*	SW:Q5E9I8	DDB1- and CUL4-associated factor 11	64.9%
1e-81	*M. musculus*	SW:Q91VU6	DDB1- and CUL4-associated factor 11	71.8%

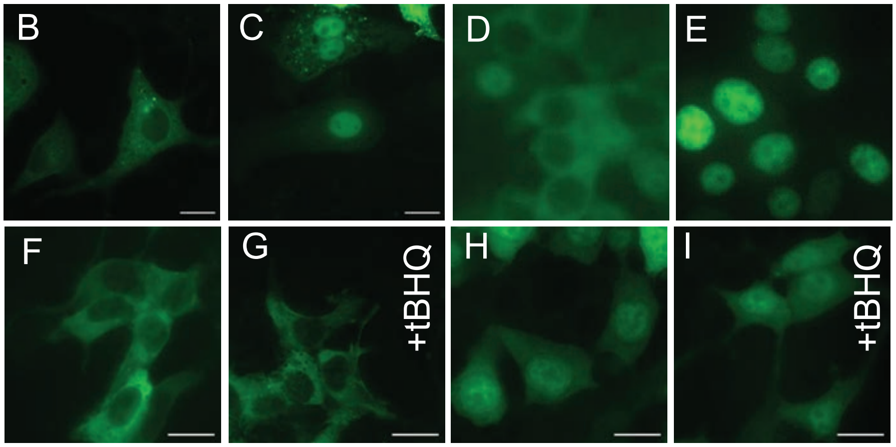

Lo and Curran
Figure S1
