# Supplementary figures and images for: WDR23 regulates NRF2 independently of KEAP1
Source: PLoS Genet. 2017 Apr 28;13(4):e1006762. doi: 10.1371/journal.pgen.1006762 (PMC5428976; doi:10.1371/journal.pgen.1006762)

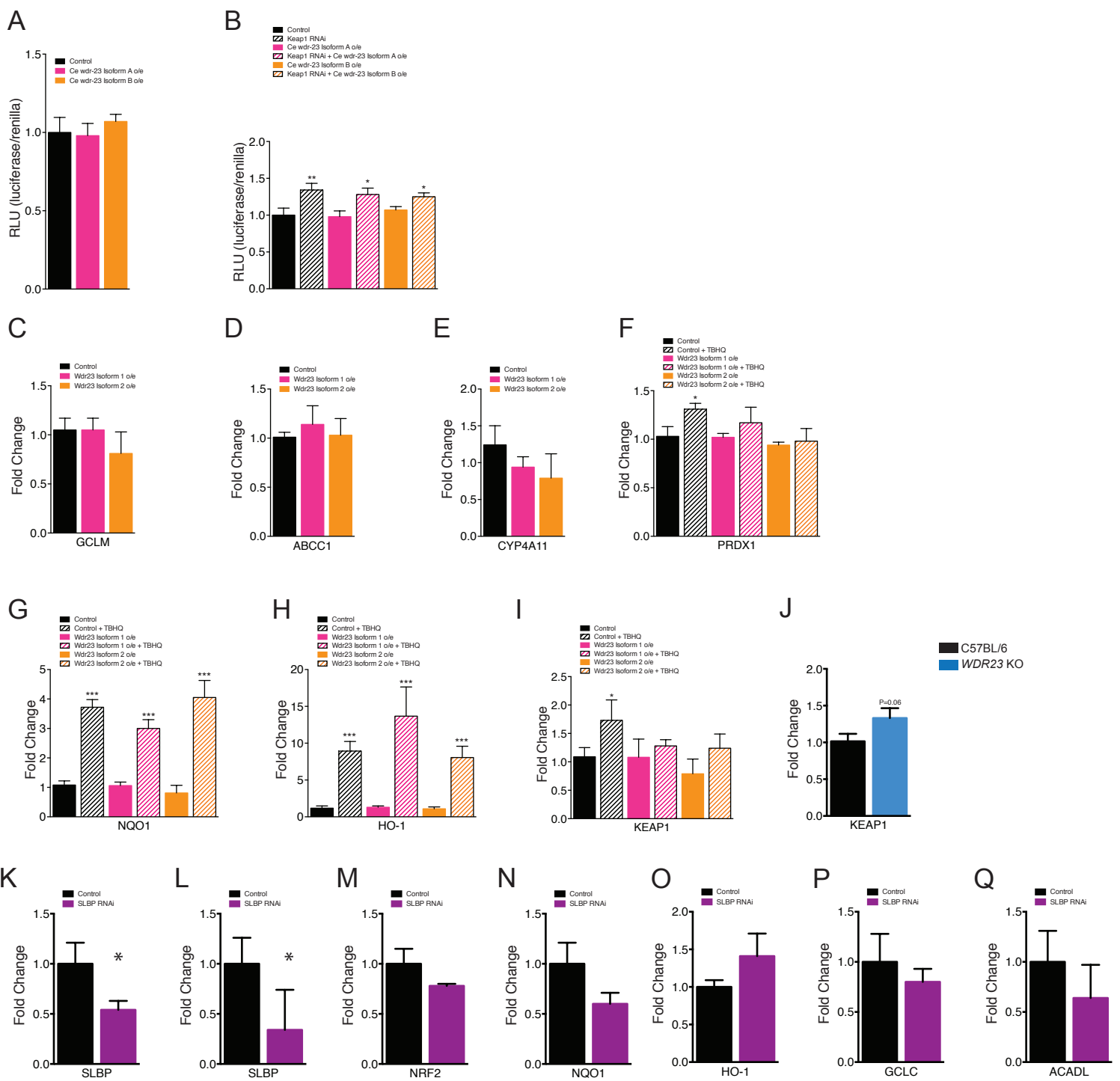

Supplement: S2 Fig — (A,B) Overexpression of C. elegans WDR-23A or WDR23B is unable to alter NRF2 transcriptional responses in normal cells (Control n = 16, Ce WDR-23A n = 16, Ce WDR-23B n = 16) (A) or in cells with reduced KEAP1 expression following KEAP1 siRNA treatment (Control n = 16, KEAP1 RNAi n = 16, Ce WDR-23A n = 16, KEAP1 RNAi + Ce WDR-23A n = 16, Ce WDR-23B n = 16, KEAP1 RNAi + Ce WDR-23B n = 15) (B). (C-E) Overexpression of WDR23 isoform 1 or isoform 2 does not significantly reduce the expression of the NRF2 targets GCLM (Control n = 9, Iso 1 n = 6, Iso 2 n = 3) (C), ABCC1 (Control n = 9, Iso 1 n = 6, Iso 2 n = 3) (D) or CYP4A11 (Control n = 9, Iso 1 n = 6, Iso 2 n = 3) (E) in the absence of stress. (F-H) The increased expression of NRF2 targets following exposure to tBHQ does not occur for PRDX1 (Control n = 9, +tBHQ n = 9, Iso 1 n = 6, Iso 1 +tBHQ n = 6, Iso 2 n = 3, Iso 2 +tBHQ n = 3) (F) when WDR23 is ectopically expressed while expression of NQO1 (Control n = 9, +tBHQ n = 9, Iso 1 n = 6, Iso 1 +tBHQ n = 6, Iso 2 n = 3, Iso 2 +tBHQ n = 3) (G) and HO-1 (Control n = 9, +tBHQ n = 9, Iso 1 n = 6, Iso 1 +tBHQ n = 6, Iso 2 n = 3, Iso 2 +tBHQ n = 3) (H) is still increased. (I) The compensatory increased expression of KEAP1 following stress is attenuated when WDR23 isoform 1 or isoform 2 are overexpressed. (Control n = 9, +tBHQ n = 9, Iso 1 n = 6, Iso 1 +tBHQ n = 6, Iso 2 n = 3, Iso 2 +tBHQ n = 3) (J) Wdr23 knockout (KO) MEF cells display increased expression of Keap1 (Control n = 5, KO n = 12). (K,L) Slbp siRNA treatment does not significantly alter transcript levels of (M) NRF2 or the NRF2 transcriptional targets (N) Nqo1, (O) Ho-1, (P) Gclc, or (Q) Acadl in HEK293T cells. (Control n = 4, Slbp RNAi n = 3). (PDF) [file pgen.1006762.s002.pdf]

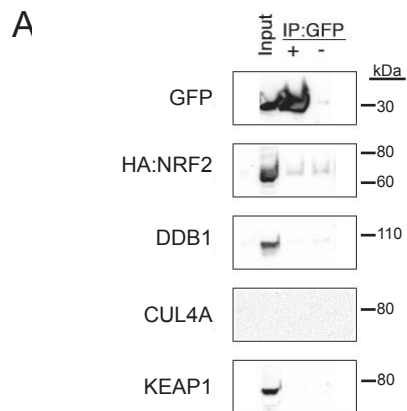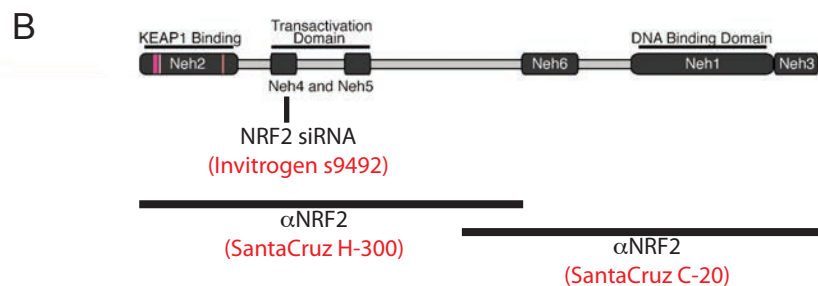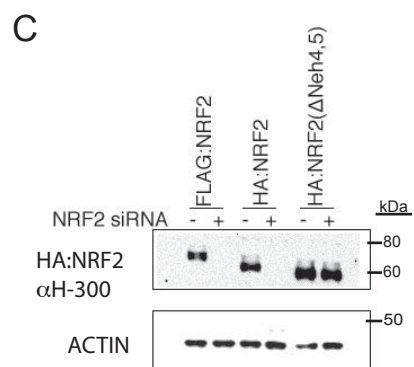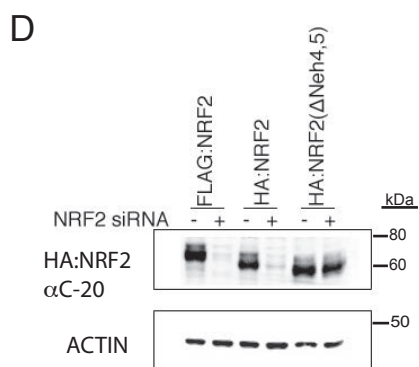

**Lo and Curran**  
**Figure S3**

Supplement: S3 Fig — (A) Overexpression of GFP and subsequent IP of the GFP protein does not pull down NRF2, DDB1, CUL4A, or KEAP1. (B) Schematic NRF2 protein and localization of the binding site of a NRF2 siRNA and domains used as antigens H-300 and C-20 for the production of NRF2 specific antibodies. (C,D) Specificity of the NRF2 protein detected in co-IPs when detected by H-300 (C) and C-20 (D). (PDF) [file pgen.1006762.s003.pdf]

A

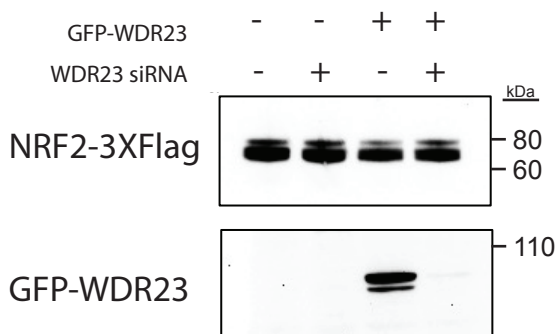

B

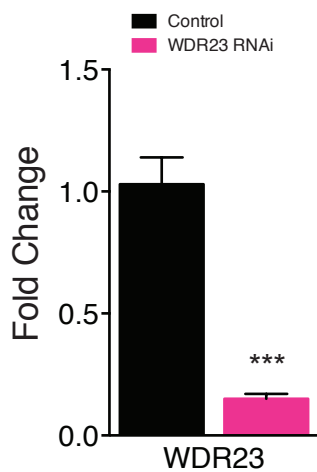

C

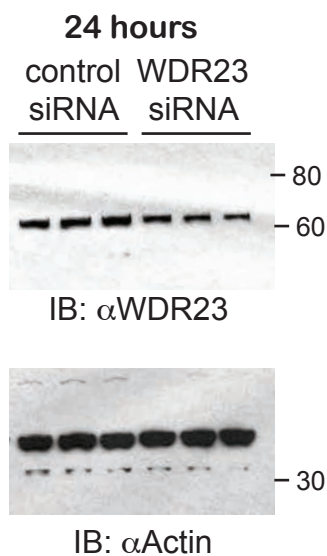

D

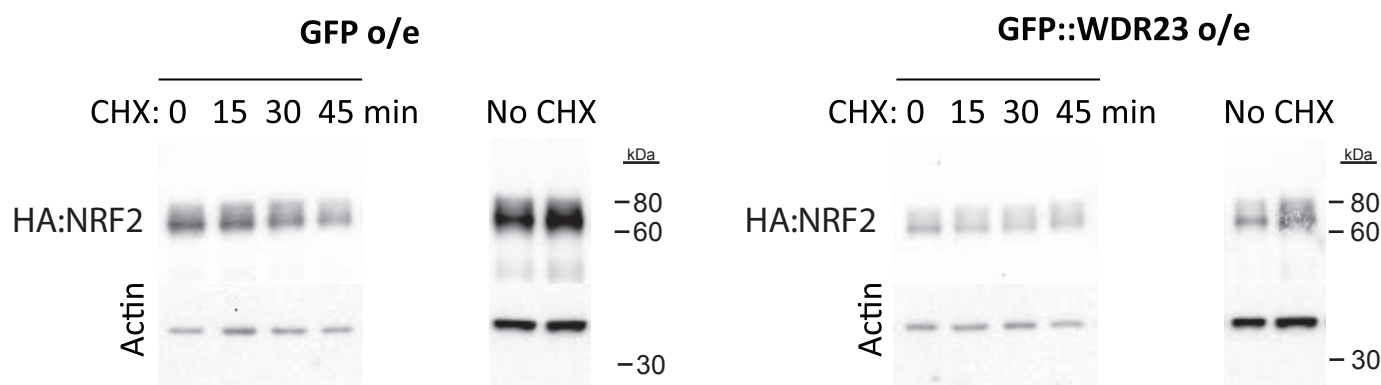

E

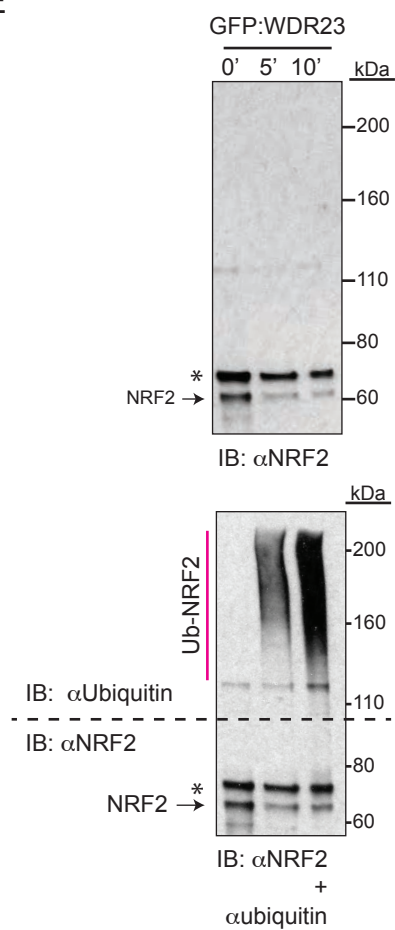

Supplement: S4 Fig — (A-C) The decreased level of NRF2 protein when WDR23 is expressed is dependent on WDR23 and reversed by WDR23 siRNA treatment (A), which efficiently reduces WDR23 mRNA levels (B), and also reduces endogenous WDR23 protein (C). (D) NRF2 protein levels are reduced when WDR23 is expressed; however a significant change in turnover rate is not detected when cells are treated with cyclohexamide (CHX). (E) Time dependent polyubiquitination of NRF2 by the CUL4-DDB1-WDR23 complex. *, non-specific cross reacting band. (PDF) [file pgen.1006762.s004.pdf]

A

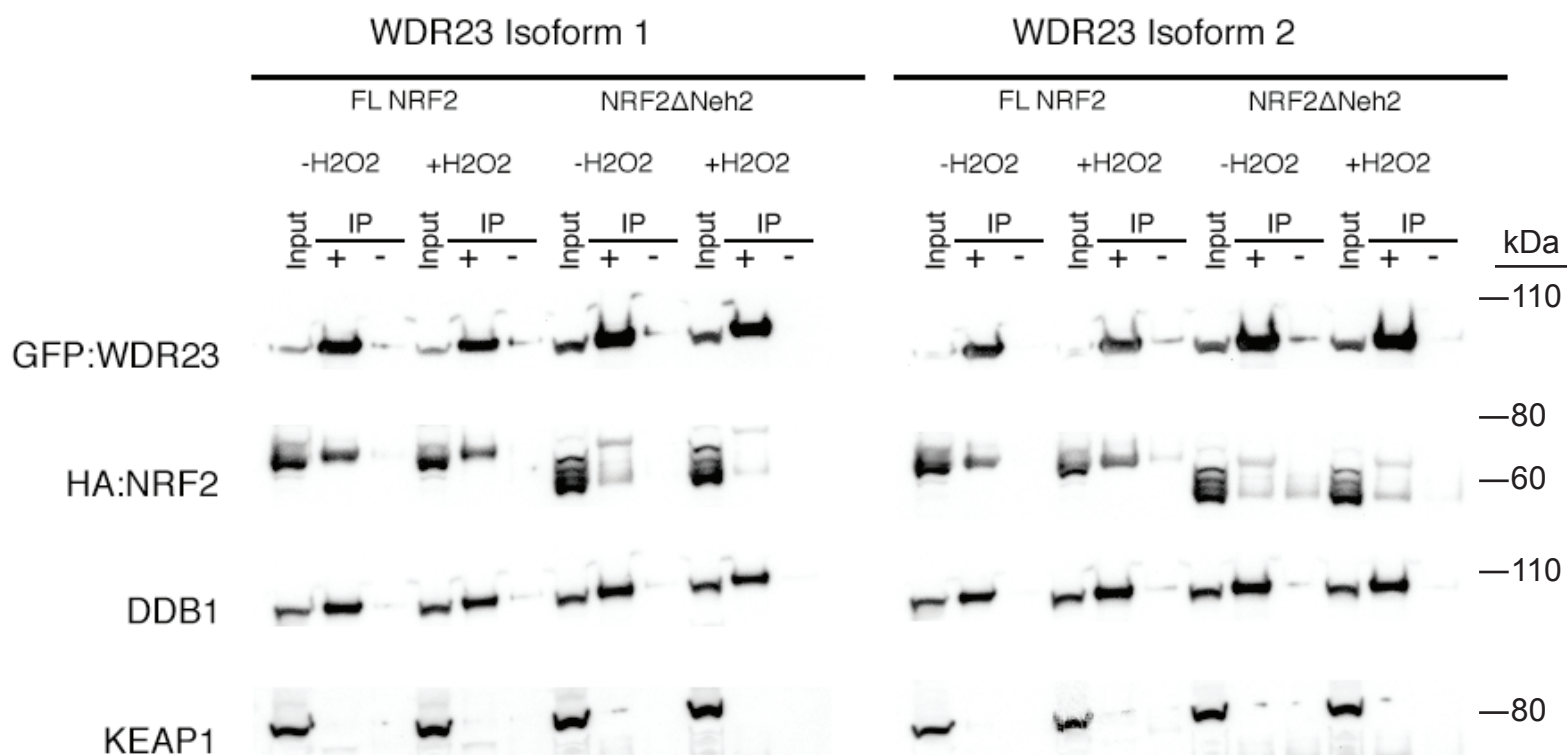

Lo and Curran  
Figure S5

Supplement: S5 Fig — (A) The interaction of WDR23 isoform 1 or WDR23 isoform 2 with NRF2 occurs even in the presence of oxidative stress. (PDF) [file pgen.1006762.s005.pdf]

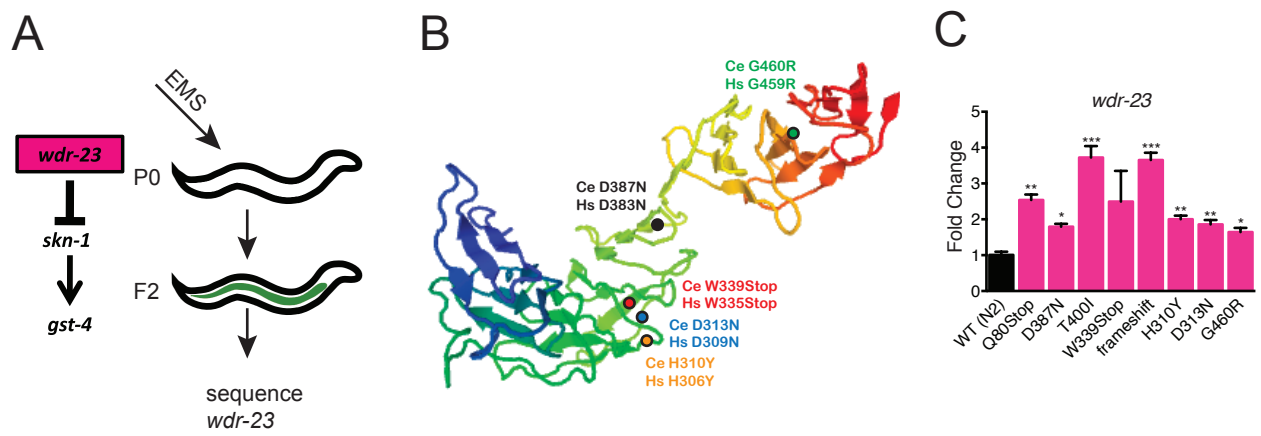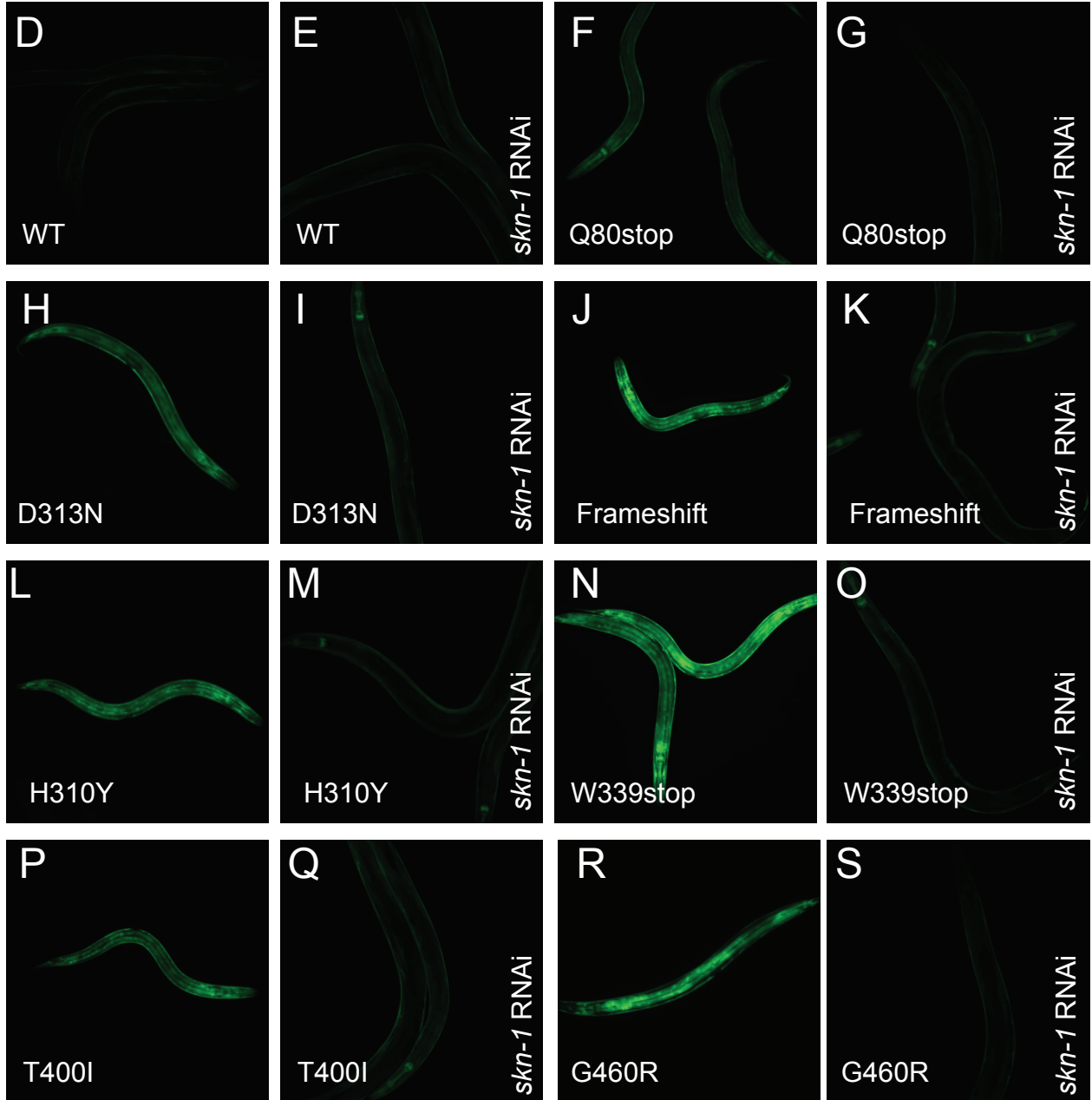

Lo and Curran  
Figure S6

Supplement: S6 Fig — (A) Schematic of EMS mutagenesis screen to identify wdr-23 mutations, which lead to activation of the SKN-1 reporter gst-4::gfp. (B) Location of conserved mutations in the crystal structure of DCAF provided by the Protein Model Portal[61]. (C) Strains harboring mutant versions of WDR-23 display compensatory increased expression of wdr-23 itself. (WT n = 3, Q80Stop n = 3, D387N n = 3, T400I n = 3, W339Stop n = 3, frameshift n = 3, H310Y n = 3, D313N n = 3, G460R n = 3). (D-S) The increased expression of the SKN-1/NRF2 transcriptional reporter gst-4::gfp in wdr-23 mutants (D,F,H,J,L,N,P,R) is dependent on skn-1 (E,G,I,K,M,O,Q,S). (PDF) [file pgen.1006762.s006.pdf]

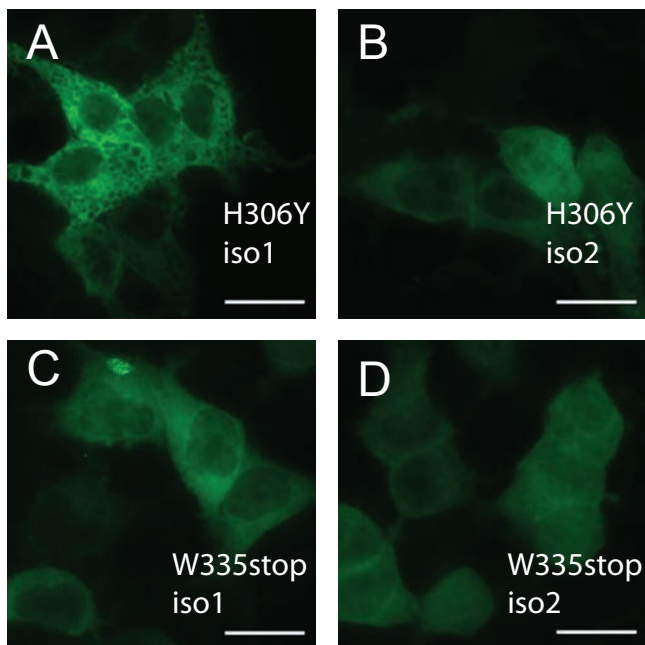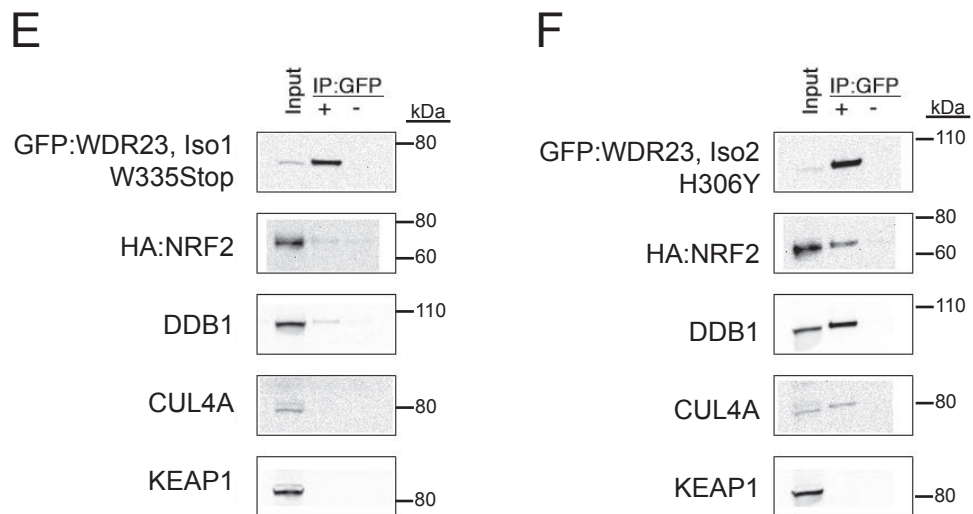

Lo and Curran

Figure S7

Supplement: S7 Fig — (A-D) The H306Y (A,B) and W335Stop (C,D) mutations do not measurably change the subcellular localization of WDR23 isoform 1 while the same mutations in WDR23 isoform 2 leads to more cytoplasmic protein. (E) The W335Stop mutation in WDR23 isoform 1 reduces the interaction with NRF2 and DDB1-CUL4 complexes. (F) The H306Y mutation in WDR23 isoform 2 modestly reduces the interaction with NRF2 and DDB1-CUL4. (PDF) [file pgen.1006762.s007.pdf]

A

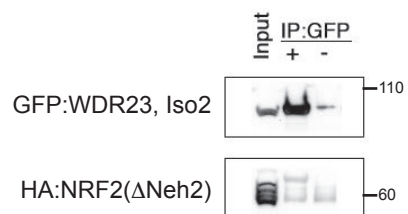

B

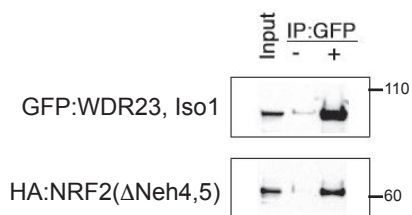

C

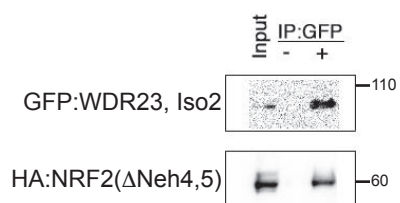

D

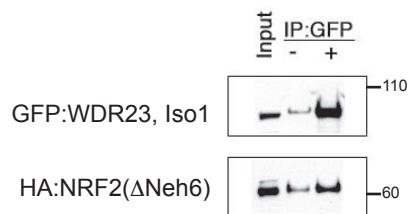

E

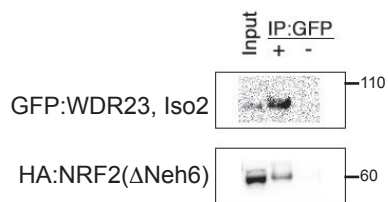

F

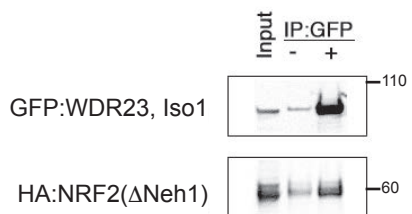

G

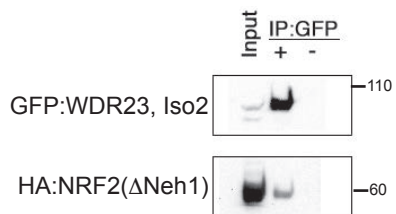

H

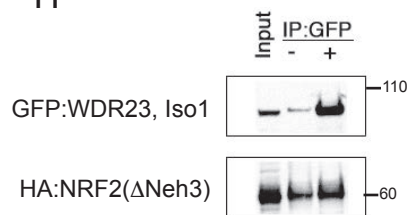

I

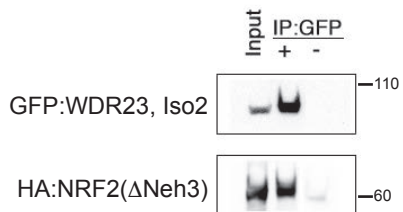

Supplement: S8 Fig — (A) WDR23 isoform 2 does not interact with NRF2ΔNeh2. (B-I) WDR23 isoform 1 (B,D,F,H) or WDR23 isoform 2 (C,E,G,I) still interact with NRF2ΔNeh4,5 (B,C), NRF2ΔNeh6 (D,E), NRF2ΔNeh1 (F,G), and NRF2ΔNeh3 (H,I). (PDF) [file pgen.1006762.s008.pdf]

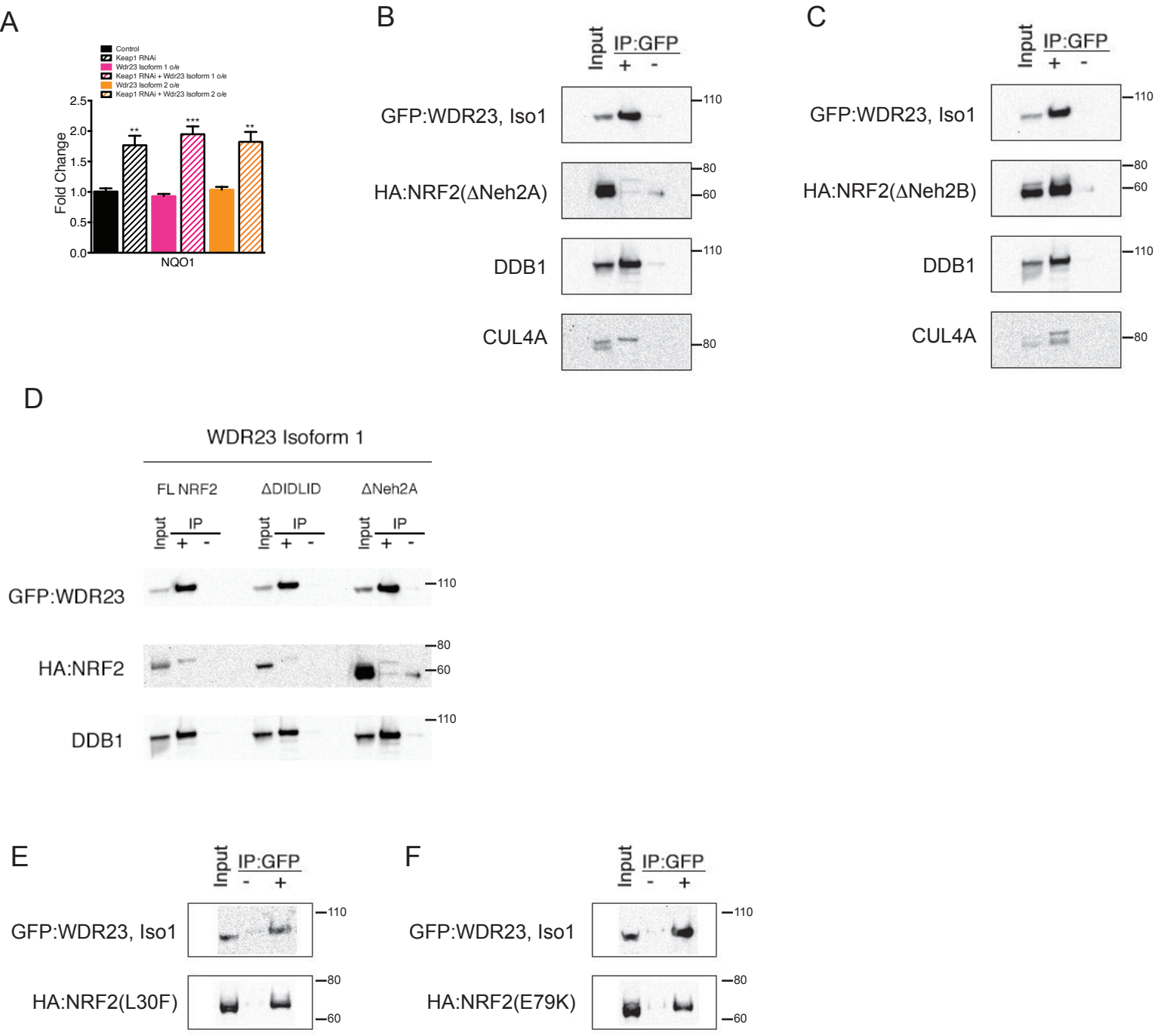

Supplement: S9 Fig — (A) Although overexpression of WDR23 can suppress some NRF2 transcriptional targets when KEAP1 is reduced, NQO1 expression remains high. (Control n = 6, KEAP1 RNAi n = 6, Iso 1 n = 6, Iso 1 + KEAP1 n = 6, Iso 2 n = 6, Iso 2 + KEAP1 RNAi n = 6). (B-C) Deletion of the first half of the Neh2 domain (ΔNeh2A) abolishes binding to WDR23 (B), while deletion of the second half of the Neh2 domain (ΔNeh2B) can still interact (C). (D) Single blot assessment of differential binding of WDR23 to full length NRF2, ΔNeh2A, and ΔDIDLID. (E,F) Mutation of the DLG (E) or ETGE (F) motifs in the Neh2 domain of NRF2 does not abolish binding by WDR23. (PDF) [file pgen.1006762.s009.pdf]

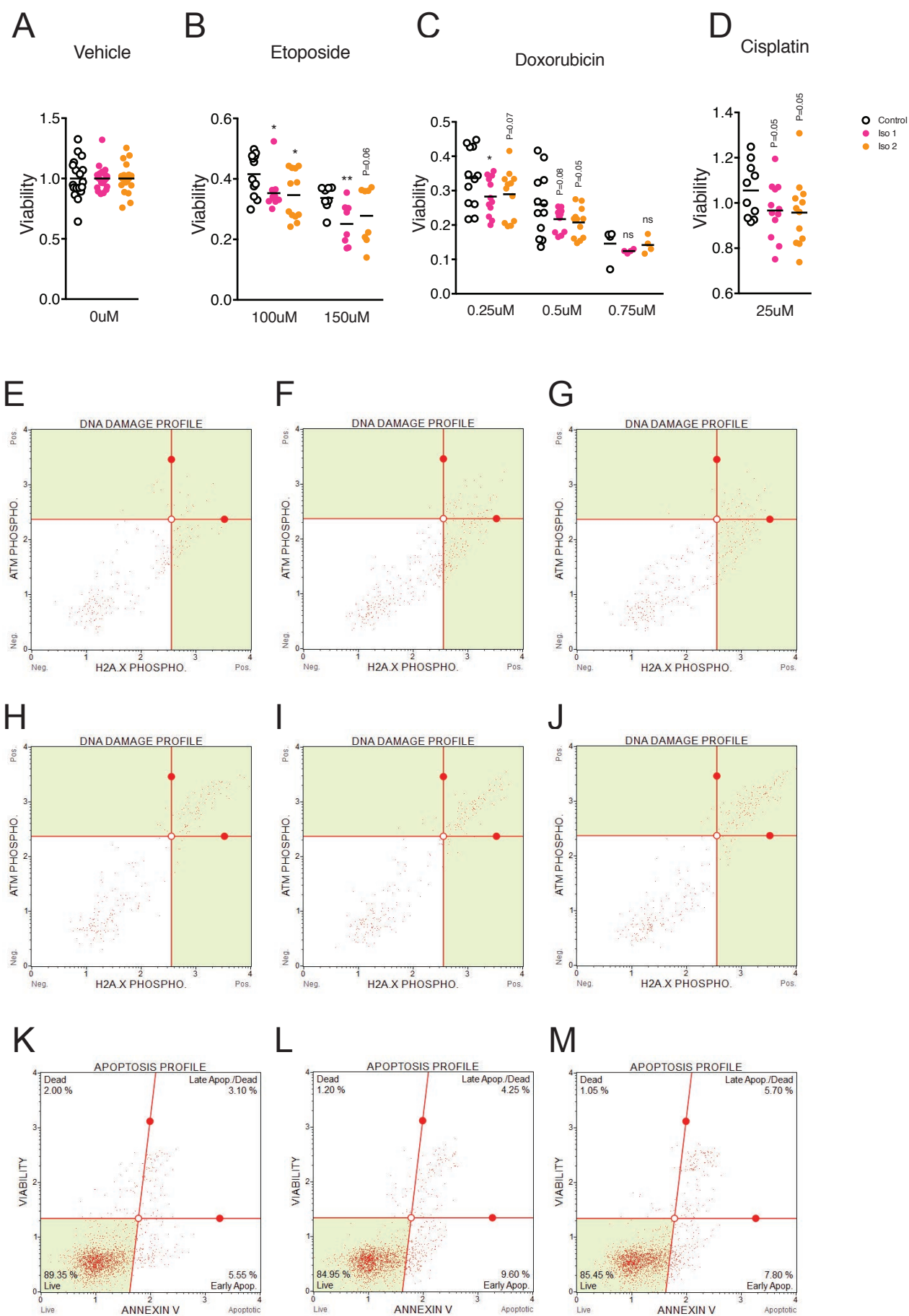

Supplement: S10 Fig — (A) Viability of cells with treatment of the vehicle DMSO. (Control n = 18, Iso 1 n = 20, Iso 2 n = 20) (B-D) Cells overexpressing GFP:WDR23 isoform 1 (pink) or GFP:WDR23 isoform 2 (orange) are more sensitive to etoposide (100uM n = 12 each, 150uM n = 8 each) (B), doxorubicin (0.25uM n = 12 each, 0.5uM n = 12 each, 0.75uM n = 4 each) (C), and cisplatin (Control n = 10, Iso 1 n = 12, Iso 2 n = 12) (D). (E-J) Cells overexpressing GFP:WDR23 isoform 1 (F) or GFP:WDR23 isoform 2 (G) have increased overall levels of DNA double-stranded breaks compared to cells overexpressing GFP alone (E), and upon treatment with etoposide, cells overexpressing GFP:WDR23 isoform 1 (I) or GFP:WDR23 isoform 2 (J) results in enhanced DNA double-stranded breaks compared to cells overexpressing GFP alone (H). (K-M) Cells overexpressing GFP:WDR23 isoform 1 (L) or GFP:WDR23 isoform 2 (M) have increased number of apoptotic cells compared to cells overexpressing GFP alone (K). Data are mean ± s.e.m.; one-tailed t-test relative to control GFP overexpression for each treatment condition. (PDF) [file pgen.1006762.s010.pdf]
