# Supplementary material for: WDR23 regulates NRF2 independently of KEAP1
Source: PLoS Genet. 2017 Apr 28;13(4):e1006762. doi: 10.1371/journal.pgen.1006762 (PMC5428976; doi:10.1371/journal.pgen.1006762)
Supplement: S11 Fig — (A) Location of WDR23 mutations sequence-confirmed from somatic tumors in human patients. Pink lines are in regions identified in C. elegans and human in this study that result in SKN-1/NRF2 activation. (B) Somatic tumors isolated from lung cancer patients with confirmed KEAP1 mutations have increased expression of WDR23. (C) Samples from stomach cancers with confirmed WDR23 mutations have increased expression of KEAP1. (D,E) Total NRF2 protein is reduced in A549 cells transiently transfected with WDR23 isoform 1 or isoform 2; quantified in (E). (F) Increased expression of GFP:WDR23 isoform 1 or isoform 2 can deplete activated endogenous NRF2 (Alexa Fluor 594, red) from the nucleus (DAPI, blue) in H460 cells. Scale bar, 20um. (G) Quantification of nuclear or diffuse localization of endogenous NRF2 in H460 cells overexpressing GFP:WDR23 isoform 1 (n = 188), GFP:WDR23 isoform 2 (n = 130), or GFP (n = 225) from the same experiment shown. Each bar represents an independent immunostaining experiment. Fisher’s exact test; **P<0.01, ***P<0.001. (PDF) [file pgen.1006762.s011.pdf]

A

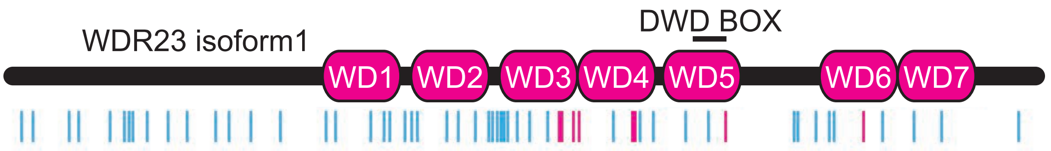

B

Sample	KEAP1 Mutation	CDS Mutation	Tissue	Fold WDR23
TCGA-46-6025-01	p.Q75*	c.223C>T	Lung	-2.51
TCGA-91-6848-01	p.D236N	c.706G>A	Lung	-2.34
TCGA-35-5375-01	p.E593fs*2	c.1777delG	Lung	CNV 4.21
TCGA-49-4488-01	p.Q284L	c.851A>T	Lung	2.06
TCGA-4B-A93V-01	p.L199F	c.597G>T	Lung	4.48
TCGA-55-A4DF-01	p.?	c.1708+1G>T	Lung	3.01
TCGA-73-4670-01	p.V123L	c.367G>T	Lung	2.08
TCGA-73-4677-01	p.K287*	c.859A>T	Lung	3.63
TCGA-86-8358-01	p.G558E	c.1673G>A	Lung	3.58
TCGA-86-A4D0-01	p.G480W	c.1438G>T	Lung	2.53
TCGA-43-6143-01	p.I506V	c.1516A>G	Lung	3.02
TCGA-44-7667-01	p.?	c.1709-1G>T	Lung	4.84

C

Sample	WDR23 Mutation	CDS Mutation	Tissue	Fold KEAP1
TCGA-78-7220-01	p.R145L	c.434G>T	Lung	2.07
TCGA-BR-8361-01	p.R341W	c.1021C>T	Stomach	2.43
TCGA-BR-8372-01	p.Q70R	c.209A>G	Stomach	2.15
TCGA-CG-5721-01	p.R265H	c.794G>A	Stomach	3.41
TCGA-BR-8361-01	p.R341W	c.1021C>T	Stomach	2.43

D

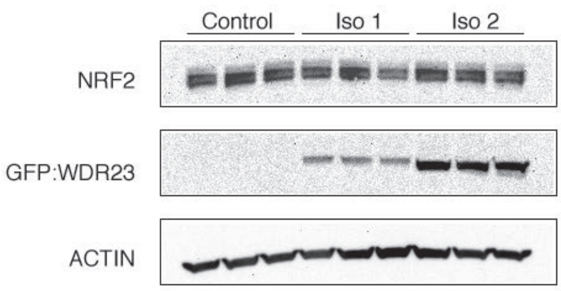

E

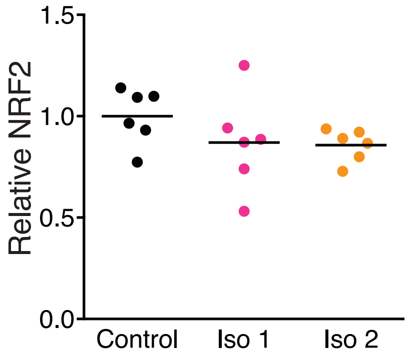

F

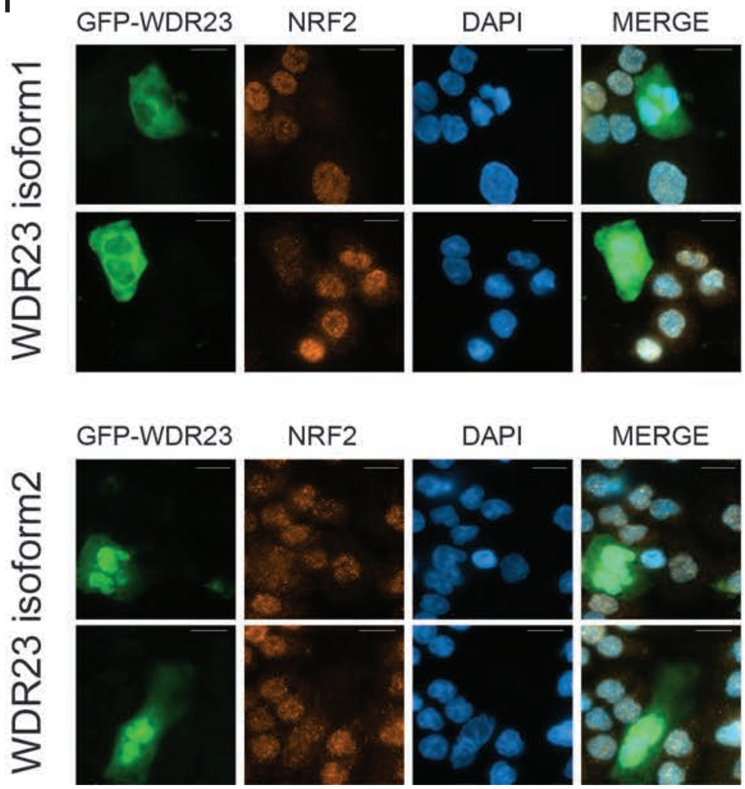

G

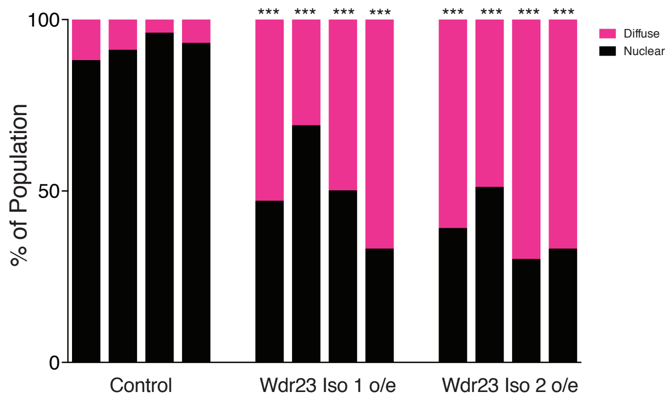

Lo and Curran
Figure S11
