# Supplementary material for: WDR23 regulates NRF2 independently of KEAP1
Source: PLoS Genet. 2017 Apr 28;13(4):e1006762. doi: 10.1371/journal.pgen.1006762 (PMC5428976; doi:10.1371/journal.pgen.1006762)
Supplement: S1 Table — (PDF) [file pgen.1006762.s012.pdf]

S1 Table. RNAi efficiencies

Genotype	siRNA	Average Fold Change[1]	S.E.M.	P-value[2]
Wild-type	*WDR23*	0.147	0.018	***
Wild-type	*NRF2*	0.548	0.047	***
Wild-type	*KEAP1*	0.649	0.054	***
WDR23 Iso 1 o/e	*KEAP1*	0.531	0.025	***
WDR23 Iso 2 o/e	*KEAP1*	0.590	0.046	***

[1] As compared to wild-type with control siRNA
[2] *P<0.05, **P<0.01, ***P<0.001
