# Supplementary material for: WDR23 regulates NRF2 independently of KEAP1
Source: PLoS Genet. 2017 Apr 28;13(4):e1006762. doi: 10.1371/journal.pgen.1006762 (PMC5428976; doi:10.1371/journal.pgen.1006762)
Supplement: S2 Table — (PDF) [file pgen.1006762.s013.pdf]

S2 Table. *C. elegans wdr-23* mutants

Strain	Allele	Amino Acid Mutation
SPC296	*lax101*	Q80Stop
SPC318	*lax123*	D387N
SPC302	*lax124*	T400I
SPC206	*lax126*	W339Stop
SPC299	*lax129*	Frameshift, early stop
SPC315	*lax134*	H310Y
SPC303	*lax211*	D313N
SPC317	*lax213*	G460R
