# Supplementary material for: WDR23 regulates NRF2 independently of KEAP1
Source: PLoS Genet. 2017 Apr 28;13(4):e1006762. doi: 10.1371/journal.pgen.1006762 (PMC5428976; doi:10.1371/journal.pgen.1006762)
Supplement: S3 Table — (PDF) [file pgen.1006762.s014.pdf]

S3 Table . DWD-box motif homology

DWD-consensus	sssxDxxhxhWDhR
H. sapiens	SNSKDQTIKLWDIR
M. musculus	SNSKDQTIKLWDIR
A. melanoleuca	SNSKDQTIKLWDIR
T. guttata	SGSLDKTIRLWDLR
D. melanogaster	SNSKDQSIKIWDIR
S. pombe	SASSDGEVKLWDIR
S. cerevisiae	SSSKDGTIKIWDTV
