# Supplementary material for: WDR23 regulates NRF2 independently of KEAP1
Source: PLoS Genet. 2017 Apr 28;13(4):e1006762. doi: 10.1371/journal.pgen.1006762 (PMC5428976; doi:10.1371/journal.pgen.1006762)
Supplement: S4 Table — (PDF) [file pgen.1006762.s015.pdf]

S4 Table. Raw MTT cell survival assay data

Experiment 1

Genotype	Treatment	A540	Average	SEM	Relative	Average	SEM
Control	Control		2.2538	0.4070775		1	0.1806183
		2.5553			1.1337741		
		1.4481			0.6425149		
		2.758			1.2237111		
Control	25uM Cisplatin	2.7057	2.66135	0.0628382	1.2005058	1.1808279	0.027881
		2.6012			1.1541397		
		2.5249			1.1202857		
		2.8136			1.2483805		
Control	50uM Cisplatin	2.8532	2.7522	0.1129219	1.2659508	1.2211376	0.0501029
		2.8551			1.2667939		
		2.8863			1.2806371		
		2.4142			1.0711687		
Control	25uM Etoposide	1.2957	1.31695	0.0450149	0.5748957	0.5843243	0.0199729
		1.1983			0.5316798		
		1.3863			0.6150945		
		1.3875			0.6156269		
Control	100uM Etoposide	1.0281	1.0691	0.0200478	0.4561629	0.4743544	0.0088951
		1.0536			0.4674771		
		1.0718			0.4755524		
		1.1229			0.4982252		
Control	0.25uM Doxorubicin	1.0095	0.9809	0.0114132	0.4479102	0.4352205	0.005064
		0.9643			0.4278552		
		0.9608			0.4263022		
		0.989			0.4388144		
Control	0.5uM Doxorubicin	0.9387	0.826675	0.0527621	0.4164966	0.3667916	0.0234103
		0.7454			0.3307303		
		0.8943			0.3967965		
		0.7283			0.3231431		
Control	1uM Doxorubicin	0.7686	0.726775	0.038329	0.341024	0.3224665	0.0170064
		0.8042			0.3568196		
		0.6299			0.2794835		
		0.7044			0.3125388		

Genotype	Treatment	A540	Average	SEM	Relative	Average	SEM	ttest
Isoform 1	Control	2.7779	3.185	0.1400786	0.8721821	1	0.0439807	
		3.4118			1.0712088			
		3.2432			1.0182732			
		3.3071			1.0383359			
Isoform 1	25uM Cisplatin	2.574	2.734725	0.150975	0.8081633	0.8586264	0.0474019	0.0011338
		2.9454			0.9247724			
		3.0276			0.9505808			
		2.3919			0.750989			
Isoform 1	50uM Cisplatin	2.6924	2.698325	0.0617355	0.8453375	0.8471978	0.0193832	0.0012542
		2.5336			0.7954788			
		2.7389			0.8599372			
		2.8284			0.8880377			
Isoform 1	25uM Etoposide	1.1967	1.136225	0.0381761	0.37573	0.3567425	0.0119862	0.0001053
		1.0259			0.3221036			
		1.1467			0.3600314			
		1.1756			0.3691052			
Isoform 1	100uM Etoposide	1.0536	1.07835	0.0086245	0.3308006	0.3385714	0.0027079	0.000134
		1.085			0.3406593			
		1.0814			0.339529			
		1.0934			0.3432967			
Isoform 1	0.25uM Doxorubicin	1.1372	0.8883	0.094862	0.3570487	0.2789011	0.029784	0.0060881
		0.8884			0.2789325			
		0.6771			0.2125903			
		0.8505			0.267033			
Isoform 1	0.5uM Doxorubicin	0.7521	0.742225	0.019719	0.2361381	0.2330377	0.0061912	0.0041085
		0.7211			0.226405			
		0.7028			0.2206593			
		0.7929			0.2489482			
Isoform 1	1uM Doxorubicin	0.7153	0.7409	0.0353093	0.224584	0.2326217	0.0110861	0.0031832
		0.7144			0.2243014			
		0.6887			0.2162323			
		0.8452			0.2653689			

Genotype	Treatment	A540	Average	SEM	Relative	Average	SEM	ttest
Isoform 2	Control	3.2002	3.2007	0.0606963	0.9998438	1	0.0189635	
		3.2989			1.0306808			
		3.0298			0.9466054			
		3.2739			1.02287			
Isoform 2	25uM Cisplatin	2.6959	2.7752	0.2260894	0.8422845	0.8670603	0.0706375	0.0075727
		2.3633			0.7383697			
		2.6228			0.8194457			
		3.4188			1.0681413			
Isoform 2	50uM Cisplatin	2.8087	2.7145	0.1139156	0.8775268	0.8480957	0.0355908	0.000661
		2.5804			0.8061986			
		2.4814			0.7752679			
		2.9875			0.9333896			
Isoform 2	25uM Etoposide	1.1729	1.180075	0.0091984	0.3664511	0.3686928	0.0028739	0.0007298
		1.1579			0.3617646			
		1.1902			0.3718562			
		1.1993			0.3746993			
Isoform 2	100uM Etoposide	1.3922	0.9706	0.143051	0.4349674	0.3032462	0.0446937	0.0144627
		0.8132			0.2540694			
		0.7747			0.2420408			
		0.9023			0.2819071			
Isoform 2	0.25uM Doxorubicin	0.9089	1.0055	0.033819	0.2839691	0.31415	0.0105661	0.0001675
		1.0185			0.3182116			
		1.0664			0.3331771			
		1.0282			0.3212422			
Isoform 2	0.5uM Doxorubicin	0.6981	0.70205	0.0044873	0.2181085	0.2193426	0.001402	0.0039776
		0.6955			0.2172962			
		0.7153			0.2234824			
		0.6993			0.2184835			
Isoform 2	1uM Doxorubicin	0.5675	0.6532	0.0633951	0.177305	0.2040804	0.0198066	0.0020921
		0.5536			0.1729622			
		0.6628			0.2070797			
		0.8289			0.2589746			

Genotype	Treatment	A540	Average	SEM	Relative	Average	SEM
Control	Control		2.2215667	0.2159264		1	0.0971955
		2.6407			1.1886657		
		1.9219			0.8651102		
		2.1021			0.9462241		
Control	75uM Cisplatin	2.6412	2.471875	0.0760526	1.1888907	1.112672	0.0342338
		2.2884			1.0300839		
		2.4199			1.0892763		
		2.538			1.142437		
Control	100uM Cisplatin	2.7274	2.43845	0.1300682	1.2276922	1.0976263	0.0585479
		2.525			1.1365853		
		2.3948			1.077978		
		2.1066			0.9482497		
Control	50uM Etoposide	0.9228	0.821725	0.0607193	0.4153825	0.3698854	0.0273318
		0.8793			0.3958018		
		0.8376			0.3770312		
		0.6472			0.2913259		
Control	75uM Etoposide	0.636	0.71165	0.0339019	0.2862845	0.320337	0.0152603
		0.7493			0.3372845		
		0.6762			0.3043798		
		0.7851			0.3533993		
Control	150uM Etoposide	0.6972	0.679275	0.0379616	0.3138326	0.305764	0.0170878
		0.7286			0.3279668		
		0.724			0.3258961		
		0.5673			0.2553603		
Control	0.75uM Doxorubicin	0.1582	0.3237	0.0552837	0.071211	0.145708	0.024885
		0.3689			0.166054		
		0.3821			0.1719957		
		0.3856			0.1735712		
Control	2uM Doxorubicin	0.676	0.572625	0.0404821	0.3042898	0.2577573	0.0182223
		0.5979			0.2691344		
		0.5026			0.2262367		
		0.514			0.2313683		

Genotype	Treatment	A540	Average	SEM	Relative	Average	SEM	ttest
Isoform 1	Control	2.6567	2.873525	0.0750858	0.9245439	1	0.0261302	
		2.8914			1.0062206			
		2.9893			1.0402902			
		2.9567			1.0289453			
Isoform 1	75uM Cisplatin	2.2871	2.44125	0.1113466	0.7959214	0.8495663	0.0387491	0.0011742
		2.5355			0.8823657			
		2.2321			0.7767811			
		2.7103			0.9431969			
Isoform 1	100uM Cisplatin	2.2662	2.23435	0.0168871	0.7886481	0.7775641	0.0058768	0.0057908
		2.1998			0.7655406			
		2.2603			0.7865949			
		2.2111			0.769473			
Isoform 1	50uM Etoposide	0.7061	0.692575	0.0172987	0.2457261	0.2410193	0.00602	0.0078465
		0.6779			0.2359123			
		0.7331			0.2551222			
		0.6532			0.2273166			
Isoform 1	75uM Etoposide	0.6491	0.641375	0.0231963	0.2258898	0.2232015	0.0080724	0.0016475
		0.5736			0.1996155			
		0.6708			0.2334415			
		0.672			0.2338591			
Isoform 1	150uM Etoposide	0.4909	0.54355	0.0309976	0.1708355	0.1891579	0.0107873	0.0010535
		0.5649			0.1965878			
		0.4967			0.1728539			
		0.6217			0.2163545			
Isoform 1	0.75uM Doxorubicin	0.3572	0.356525	0.0076536	0.1243073	0.1240724	0.0026635	0.2248052
		0.3369			0.1172428			
		0.3743			0.1302581			
		0.3577			0.1244813			
Isoform 1	2uM Doxorubicin	0.4771	0.49425	0.0192868	0.166033	0.1720013	0.0067119	0.0064764
		0.4895			0.1703483			
		0.461			0.1604301			
		0.5494			0.1911937			

Genotype	Treatment	A540	Average	SEM	Relative	Average	SEM	ttest
Isoform 2	Control	2.9508	2.871275	0.1429469	1.0276968	1	0.0497852	
		2.8093			0.9784155			
		2.5198			0.8775892			
		3.2052			1.1162985			
Isoform 2	75uM Cisplatin	2.4384	2.451975	0.0973417	0.8492394	0.8539673	0.0339019	0.0008565
		2.3785			0.8283776			
		2.2671			0.7895795			
		2.7239			0.9486726			
Isoform 2	100uM Cisplatin	2.1973	2.2644	0.0781111	0.7652698	0.7886392	0.0272043	0.0037809
		2.3947			0.8340197			
		2.0751			0.7227103			
		2.3905			0.832557			
Isoform 2	50uM Etoposide	0.742	0.77005	0.0387808	0.2584218	0.268191	0.0135065	0.0126057
		0.7488			0.2607901			
		0.7064			0.2460231			
		0.883			0.3075289			
Isoform 2	75uM Etoposide	0.6754	0.66255	0.0353305	0.2352265	0.2307511	0.0123048	0.0021283
		0.7288			0.2538245			
		0.5626			0.1959408			
		0.6834			0.2380127			
Isoform 2	150uM Etoposide	0.5697	0.55225	0.0519747	0.1984136	0.1923362	0.0181016	0.0019485
		0.6409			0.2232109			
		0.5957			0.2074688			
		0.4027			0.1402513			
Isoform 2	0.75uM Doxorubicin	0.3351	0.40715	0.0353523	0.1167077	0.1418011	0.0123124	0.4471624
		0.3742			0.1303254			
		0.4997			0.1740342			
		0.4196			0.1461372			
Isoform 2	2uM Doxorubicin	0.4728	0.48525	0.0277403	0.1646655	0.1690016	0.0096613	0.0047203
		0.5205			0.1812784			
		0.4123			0.1435947			
		0.5354			0.1864677			

S4 Table – cont.

Experiment 2

Genotype	Treatment	A540	Average	SEM	Relative	Average	SEM	
Control	Control	3.8911	2.9365	0.3246975	1.3250809	1	0.110573	
		2.4357			0.8294568			
		2.7171			0.9252852			
		2.7021			0.9201771			
Control	25uM Cisplatin		2.7824333	0.0786395		0.9475339	0.02678	
		2.7189			0.9258982			
		2.6896			0.9159203			
		2.9388			1.0007832			
Control	50uM Cisplatin	3.0213	2.741775	0.1092953	1.0288779	0.9336881	0.0372196	
		2.6652			0.9076111			
		2.7795			0.946535			
		2.5011			0.8517282			
Control	25uM Etoposide	1.3766	1.2145	0.0737554	0.4687894	0.4135876	0.0251168	
		1.1441			0.3896135			
		1.2908			0.4395709			
		1.0465			0.3563766			
Control	100uM Etoposide	0.8786	1.16775	0.1421819	0.2991997	0.3976673	0.0484188	
		1.4253			0.4853737			
		1.3982			0.4761451			
		0.9689			0.3299506			
Control	0.25uM Doxorubicin	0.7743	0.704525	0.0376962	0.2636813	0.23992	0.0128371	
		0.6424			0.2187638			
		0.7651			0.2605483			
		0.6363			0.2166865			
Control	0.5uM Doxorubicin	0.5627	0.471475	0.0336665	0.1916227	0.1605568	0.0114648	
		0.4004			0.1363528			
		0.4579			0.1559339			
		0.4649			0.1583177			
Control	1uM Doxorubicin	0.5265	0.570375	0.0234244	0.1792951	0.1942363	0.007977	
		0.5612			0.1911119			
		0.5571			0.1897156			
		0.6367			0.2168227			

Genotype	Treatment	A540	Average	SEM	Relative	Average	SEM	ttest
Isoform 1	Control	2.6271	2.900025	0.3102741	0.9058887	1	0.1069901	
		2.5916			0.8936475			
		3.8297			1.3205748			
		2.5517			0.879889			
Isoform 1	25uM Cisplatin	3.0748	3.105	0.1417773	1.0602667	1.0706804	0.0488883	0.0423151
		3.1079			1.0716804			
		2.7723			0.9559573			
		3.465			1.1948173			
Isoform 1	50uM Cisplatin	2.7739	2.647075	0.0912483	0.956509	0.9127766	0.0314647	0.3416278
		2.7666			0.9539918			
		2.3839			0.8220274			
		2.6639			0.9185783			
Isoform 1	25uM Etoposide	1.0746	1.086775	0.0349603	0.3705485	0.3747468	0.0120552	0.1154109
		0.9984			0.3442729			
		1.1658			0.4019965			
		1.1083			0.3821691			
Isoform 1	100uM Etoposide	0.9747	1.078475	0.1487895	0.3361006	0.3718847	0.0513063	0.3636689
		0.873			0.3010319			
		1.5202			0.5242024			
		0.946			0.3262041			
Isoform 1	0.25uM Doxorubicin	0.753	0.677325	0.0391733	0.2596529	0.2335583	0.0135079	0.3722405
		0.5795			0.1998259			
		0.6505			0.2243084			
		0.7263			0.2504461			
Isoform 1	0.5uM Doxorubicin	0.5184	0.502425	0.0106366	0.1787571	0.1732485	0.0036678	0.1785706
		0.4898			0.1688951			
		0.5224			0.1801364			
		0.4791			0.1652055			
Isoform 1	1uM Doxorubicin	0.5317	0.52455	0.014127	0.1833432	0.1808778	0.0048713	0.1063629
		0.4955			0.1708606			
		0.5104			0.1759985			
		0.5606			0.1933087			

Genotype	Treatment	A540	Average	SEM	Relative	Average	SEM	ttest
Isoform 2	Control	3.5703	2.848275	0.3680011	1.2534955	1	0.1292014	
		2.1588			0.7579324			
		3.3923			1.1910016			
		2.2717			0.7975705			
Isoform 2	25uM Cisplatin	2.7819	3.06755	0.2208958	0.9766964	1.0769852	0.0775542	0.0979328
		2.9061			1.0203018			
		3.7258			1.30809			
		2.8564			1.0028526			
Isoform 2	50uM Cisplatin	3.1651	2.6998	0.1747103	1.111234	0.947872	0.061339	0.4255778
		2.3626			0.8294845			
		2.7538			0.9668308			
		2.5177			0.8839385			
Isoform 2	25uM Etoposide	0.9839	0.97805	0.0582098	0.3454372	0.3433833	0.0204369	0.0375646
		1.0661			0.3742967			
		1.0504			0.3687846			
		0.8118			0.2850146			
Isoform 2	100uM Etoposide	0.7853	0.91955	0.114395	0.2757107	0.3228445	0.0401629	0.1403357
		0.8317			0.2920013			
		1.2615			0.4428996			
		0.7997			0.2807664			
Isoform 2	0.25uM Doxorubicin	0.5634	0.5778	0.0100186	0.1978039	0.2028596	0.0035174	0.0293383
		0.5859			0.2057035			
		0.6023			0.2114613			
		0.5596			0.1964698			
Isoform 2	0.5uM Doxorubicin	0.4639	0.444975	0.0100316	0.1628705	0.1562261	0.003522	0.369212
		0.4193			0.1472119			
		0.4575			0.1606235			
		0.4392			0.1541986			
Isoform 2	1uM Doxorubicin	0.4481	0.5013	0.0198259	0.1573233	0.1760013	0.0069607	0.0683393
		0.494			0.1734383			
		0.5328			0.1870606			
		0.5303			0.1861829			

S4 Table – cont.

Experiment 3

Genotype	Treatment	A540	Average	SEM	Relative	Average	SEM
Control	Control	2.9747	2.786125	0.1185097	1.0676836	1	0.0425357
		2.4876			0.892853		
		2.9786			1.0690834		
		2.7036			0.97038		
Control	25uM Cisplatin		2.7741333	0.132775		0.9956959	0.0476558
		3.035			1.0893266		
		2.6007			0.933447		
		2.6867			0.9643142		
Control	50uM Cisplatin	2.5966	2.619275	0.144703	0.9319754	0.940114	0.051937
		2.9877			1.0723496		
		2.6126			0.9377182		
		2.2802			0.8184127		
Control	25uM Etoposide	1.2813	1.2289	0.0198408	0.459886	0.4410786	0.0071213
		1.2347			0.4431603		
		1.211			0.4346539		
		1.1886			0.426614		
Control	100uM Etoposide	1.0474	1.044675	0.0334957	0.3759343	0.3749563	0.0120223
		1.1113			0.3988694		
		1.0675			0.3831486		
		0.9525			0.3418727		
Control	0.25uM Doxorubicin	0.9577	0.9305	0.0224168	0.3437391	0.3339764	0.0080459
		0.9289			0.3334021		
		0.8679			0.3115079		
		0.9675			0.3472565		
Control	0.5uM Doxorubicin	0.7468	0.69775	0.0247671	0.2680425	0.2504374	0.0088894
		0.734			0.2634483		
		0.6563			0.2355601		
		0.6539			0.2346987		
Control	1uM Doxorubicin	0.9698	0.7689	0.0710804	0.348082	0.2759747	0.0255123
		0.7692			0.2760824		
		0.6646			0.2385392		
		0.672			0.2411952		

Genotype	Treatment	A540	Average	SEM	Relative	Average	SEM	ttest
Isoform 1	Control	2.8579	2.707625	0.0530336	1.0555007	1	0.0195868	
		2.6662			0.9847006			
		2.6952			0.9954111			
		2.6112			0.9643876			
Isoform 1	25uM Cisplatin	2.8969	2.627525	0.124143	1.0699044	0.9704169	0.0458494	0.3594351
		2.6818			0.9904621			
		2.6346			0.9730299			
		2.2968			0.8482711			
Isoform 1	50uM Cisplatin	2.3776	2.3856	0.0422563	0.8781127	0.8810674	0.0156064	0.1723999
		2.462			0.909284			
		2.2702			0.838447			
		2.4326			0.8984257			
Isoform 1	25uM Etoposide	1.0081	1.013725	0.0030587	0.3723189	0.3743964	0.0011297	0.0010943
		1.0224			0.3776003			
		1.0116			0.3736116			
		1.0128			0.3740548			
Isoform 1	100uM Etoposide	0.9885	0.94255	0.0260968	0.3650801	0.3481095	0.0096382	0.0672139
		0.8804			0.3251558			
		0.9185			0.3392272			
		0.9828			0.3629749			
Isoform 1	0.25uM Doxorubicin	0.9024	0.90805	0.0180178	0.333281	0.3353677	0.0066545	0.4492497
		0.9395			0.3469831			
		0.8595			0.3174369			
		0.9308			0.3437699			
Isoform 1	0.5uM Doxorubicin	0.6851	0.662175	0.014669	0.2530262	0.2445593	0.0054177	0.2984505
		0.6869			0.253691			
		0.6259			0.231162			
		0.6508			0.2403582			
Isoform 1	1uM Doxorubicin	0.6538	0.686075	0.0310801	0.2414662	0.2533863	0.0114787	0.2314956
		0.6547			0.2417986			
		0.6565			0.2424634			
		0.7793			0.2878168			

Genotype	Treatment	A540	Average	SEM	Relative	Average	SEM	ttest
Isoform 2	Control	2.6776	2.2919	0.1391063	1.1682883	1	0.0606947	
		2.294			1.0009163			
		2.1622			0.9434094			
		2.0338			0.887386			
Isoform 2	25uM Cisplatin	2.2009	2.125975	0.1068774	0.960295	0.9276037	0.0466327	0.1781208
		2.0328			0.8869497			
		1.8875			0.8235525			
		2.3827			1.0396178			
Isoform 2	50uM Cisplatin	2.3431	2.136575	0.1027358	1.0223395	0.9322287	0.0448256	0.4561621
		2.2829			0.9960731			
		1.9732			0.8609451			
		1.9471			0.8495571			
Isoform 2	25uM Etoposide	1.2348	1.016875	0.0958929	0.538767	0.4436821	0.0418399	0.477373
		1.1211			0.4891575			
		0.8487			0.3703041			
		0.8629			0.3764998			
Isoform 2	100uM Etoposide	1.0148	0.946875	0.0351713	0.4427767	0.4131398	0.0153459	0.0503134
		0.882			0.3848335			
		0.8905			0.3885423			
		1.0002			0.4364065			
Isoform 2	0.25uM Doxorubicin	0.9521	0.80485	0.0531141	0.4154195	0.3511715	0.0231747	0.2623852
		0.7997			0.3489245			
		0.7658			0.3341333			
		0.7018			0.3062088			
Isoform 2	0.5uM Doxorubicin	0.6202	0.565825	0.0421588	0.2706052	0.2468803	0.0183947	0.4348104
		0.6303			0.275012			
		0.5662			0.2470439			
		0.4466			0.1948602			
Isoform 2	1uM Doxorubicin	0.5299	0.518025	0.0086185	0.2312055	0.2260243	0.0037604	0.072158
		0.533			0.2325581			
		0.4955			0.2161962			
		0.5137			0.2241372			

Genotype	Treatment	A540	Average	SEM	Relative	Average	SEM
Control	Control	3.0919	2.78305	0.1259683	1.1109754	1	0.0452627
		2.8852			1.0367043		
		2.5694			0.9232317		
		2.5857			0.9290886		
Control	75uM Cisplatin		2.4978	0.0823666		0.8975045	0.0295958
		2.6588			0.9553547		
		2.4475			0.8794308		
		2.3871			0.857728		
Control	100uM Cisplatin	2.6826	2.514875	0.0714429	0.9639065	0.9036399	0.0256707
		2.5408			0.9129552		
		2.5008			0.8985825		
		2.3353			0.8391154		
Control	50uM Etoposide	1.1259	1.1125	0.019191	0.4045562	0.3997413	0.0068957
		1.083			0.3891414		
		1.1608			0.4170964		
		1.0803			0.3881713		
Control	75uM Etoposide	1.0035	1.0441	0.0370993	0.3605756	0.3751639	0.0133305
		1.0459			0.3758107		
		0.9795			0.351952		
		1.1475			0.4123174		
Control	150uM Etoposide	1.032	1.024625	0.0055479	0.3708162	0.3681662	0.0019935
		1.0081			0.3622285		
		1.0291			0.3697742		
		1.0293			0.369846		
Control	2uM Doxorubicin	0.685	0.70845	0.0152684	0.2461328	0.2545588	0.0054862
		0.7272			0.2612961		
		0.7415			0.2664343		
		0.6801			0.2443722		

Genotype	Treatment	A540	Average	SEM	Relative	Average	SEM	ttest
Isoform 1	Control	2.6811	2.785525	0.1049882	0.9625116	1	0.0376906	
		2.7875			1.000709			
		2.5958			0.931889			
		3.0777			1.1048905			
Isoform 1	75uM Cisplatin	2.5932	2.48865	0.0586232	0.9309556	0.8934222	0.0210456	0.4580402
		2.5847			0.9279041			
		2.3664			0.8495346			
		2.4103			0.8652947			
Isoform 1	100uM Cisplatin	2.5439	2.255525	0.1087323	0.9132569	0.8097307	0.0390348	0.0492984
		2.1014			0.7544			
		2.3023			0.8265228			
		2.0745			0.7447429			
Isoform 1	50uM Etoposide	0.8861	0.920275	0.0295492	0.3181088	0.3303776	0.0106081	0.0012546
		1.0045			0.3606142			
		0.9172			0.3292737			
		0.8733			0.3135136			
Isoform 1	75uM Etoposide	0.9192	0.960425	0.0346057	0.3299917	0.3447914	0.0124234	0.0734233
		0.9193			0.3300276			
		0.94			0.3374588			
		1.0632			0.3816875			
Isoform 1	150uM Etoposide	0.8505	0.872325	0.0396671	0.3053284	0.3131636	0.0142404	0.0146983
		0.8066			0.2895684			
		0.8445			0.3031744			
		0.9877			0.3545831			
Isoform 1	2uM Doxorubicin	0.6924	0.664325	0.011319	0.2485707	0.2384918	0.0040635	0.0301923
		0.6675			0.2396317			
		0.6599			0.2369033			
		0.6375			0.2288617			

Genotype	Treatment	A540	Average	SEM	Relative	Average	SEM	ttest
Isoform 2	Control	2.6508	2.73985	0.0327	0.9674982	1	0.0119349	
		2.7315			0.9969524			
		2.7952			1.0202018			
		2.7819			1.0153476			
Isoform 2	75uM Cisplatin	2.343	2.039125	0.117867	0.8551563	0.7442469	0.0430195	0.0167295
		2.1015			0.7670128			
		1.8973			0.6924832			
		1.8147			0.6623355			
Isoform 2	100uM Cisplatin	2.0616	1.918875	0.0963485	0.75245	0.7003577	0.0351656	0.0021614
		1.8136			0.661934			
		1.7011			0.6208734			
		2.0992			0.7661733			
Isoform 2	50uM Etoposide	0.9613	1.02085	0.0266408	0.3508586	0.3725934	0.0097235	0.0338962
		0.9913			0.3618081			
		1.0734			0.3917733			
		1.0574			0.3859335			
Isoform 2	75uM Etoposide	0.9212	0.98905	0.0245462	0.3362228	0.3609869	0.008959	0.2079911
		1.0007			0.365239			
		0.9958			0.3634506			
		1.0385			0.3790353			
Isoform 2	150uM Etoposide	0.9849	0.996825	0.0078347	0.3594722	0.3638247	0.0028595	0.1323185
		0.9948			0.3630856			
		1.0195			0.3721007			
		0.9881			0.3606402			
Isoform 2	2uM Doxorubicin	0.649	0.648875	0.0146426	0.2368743	0.2368287	0.0053443	0.0299454
		0.6878			0.2510356			
		0.6172			0.2252678			
		0.6415			0.2341369			
