# Supplementary material for: WDR23 regulates NRF2 independently of KEAP1
Source: PLoS Genet. 2017 Apr 28;13(4):e1006762. doi: 10.1371/journal.pgen.1006762 (PMC5428976; doi:10.1371/journal.pgen.1006762)
Supplement: S5 Table — (PDF) [file pgen.1006762.s016.pdf]

S5 Table. COSMIC Database analysis of WDR23 mutations and expression in somatic tumors

Tissue	Point mutations ID/Tested	Copy number variation ID/Tested	Expression ID/Tested
Adrenal gland	n.d.	1/72 increased<br>n.d.	5/79 increased<br>n.d.
Bone	2/496	n.d.<br>n.d.	n.d.<br>n.d.
Breast	6/1350	8/987 increased<br>1/987 decreased	59/1092 increased<br>46/1092 decreased
CNS	1/2128	1/805 increased<br>n.d.	25/662 increased<br>59/662 decreased
Cervix	1/320	1/174 increased<br>n.d.	20/305 increased<br>n.d.
Endometrium	7/631	2/421 increased<br>n.d.	40/593 increased<br>11/593 decreased
Lymphoid	1/2139	n.d.<br>n.d.	12/216 increased<br>2/216 decreased
Kidney	7/1474	n.d<br>n.d..	17/595 increased<br>13/595 decreased
Large Intestine	18/1356	3/699 decreased<br>n.d.	20/602 increased<br>22/602 decreased
Liver	6/1611	n.d.<br>n.d.	24/359 increased<br>n.d.
Lung	8/1823	10/1102 increased<br>3/1102 decreased	57/1008 increased<br>30/1008 decreased
Oesophagus	4/791	3/108 increased<br>n.d.	4/125 increased<br>1/125 decreased
Ovary	2/831	2/721 increased<br>1/721 decreased	19/266 increased<br>38/266 decreased
Pancreas	2/1521	n.d.<br>n.d.	7/168 increased<br>2/168 decreased
Prostate	1/1019	n.d.<br>n.d.	16/491 increased<br>20/491 decreased
Skin	24/983	1/482 decreased<br>n.d.	20/467 increased<br>10/467 decreased
Soft tissue	n.d.	3/139 increased<br>n.d.	15/215 increased<br>7/215 decreased
Stomach	10/587	1/348 increased<br>n.d.	11/285 increased<br>4/285 decreased
Thyroid	1/567	n.d.<br>n.d.	15/512 increased<br>6/512 decreased
Upper aerodigestive tract	n.d.	4/467 increased<br>n.d.	19/517 increased<br>n.d.
Urinary tract	2/652	2/225 increased<br>n.d.	22/393 increased<br>8/393 decreased
