# Supplementary material for: WDR23 regulates NRF2 independently of KEAP1
Source: PLoS Genet. 2017 Apr 28;13(4):e1006762. doi: 10.1371/journal.pgen.1006762 (PMC5428976; doi:10.1371/journal.pgen.1006762)
Supplement: S6 Table — (PDF) [file pgen.1006762.s017.pdf]

S6 Table. qPCR primer sequences

Target Gene	Forward Sequence	Reverse Sequence
Human Targets		
B2M	AATGTCGGATGGATGAAACC	TCTCTCTTTCTGGCCTGGAG
ABCC1	GTTTCTCAGATCGCTCACCC	TCCACCAGAAGGTGATCCTC
ACADL	TAGTATTCATTCAGGTATTGTC	GCTCTGTCATTGCTATTG
ACADM	CTGGTGCTGTTGGATTAG	ATATTGCTTGGTGCTCTAC
ACADS	GATTGTGCTGTGAACTAC	CAACTTGAACTGGATGAC
CPT1A1	AATAAGCAGTCTCTTGATG	CACTTCTGTATCCTTCTTC
CYP1A1	CCCAGCTCAGCTCAGTACCT	GAGGCCAGAAGAAACTCCGT
CYP3A4	TTTTGTCCTACCATAAGGGCTTT	CACAGGCTGTTGACCATCAT
CYP4A11	CTCAAAGCCCTCCAGCAGT	ACCCATTTCTGAATCCGTTG
GCLC	CTGGGGAGTGATTTCTGCAT	AGGAGGGGGCTTAAATCTCA
GCLM	AATCTTGCCTCCTGCTGTGTGA	TGCGCTTGAATGTCAGGAATGC
GSR	CAAGCTGGGTGGCACTTG	TTGGAAAGCCATAATCAGCA
GSTA1	AATTCAGTTGTCGAGCCAGG	CCGTGCATTGAAGTAGTGGA
HO-1	AGGTCATCCCCTACACACCA	TGTTGGGGAAGGTGAAGAAG
KEAP1	CCAACTTCGCTGAGCAGATT	GCTGATGAGGGTCACCAGTT
NQO1	GTTGCCTGAAAAATGGGAGA	AAAAACCACCAGTGCCAGTC
NRF2	CGGTATGCAACAGGACATTG	GTTTGGCTTCTGGACTTGGA
SLBP	ACTTGCCCAGTCAGAGCATC	CGAGGAGGCAGAGCACC
SLBP	CAGTCAGAGCATCTGGAACG	GGAGCCTGGGACGGAAG
WDR23	TTCTCCCCCATTCATAGCAC	CAGCTTCTTCACAATGTGGC
C. elegans Targets		
snb-1	CCGGATAAGACCATCTTGACG	GACGACTTCATCAACCTGAGC
gcs-1	CCAATCGATTCCTTTGGAGA	TCGACAATGTTGAAGCAAGC
gst-4	GCTGAGCCAATCCGTATCAT	CCGAATTGTTCTCCATCGAC
ugt-11	CCGATTTCTGGGACTCTCAA	GGACTCCCAGGAAGTGTGAC
