# Supplementary material for: WDR23 regulates NRF2 independently of KEAP1
Source: PLoS Genet. 2017 Apr 28;13(4):e1006762. doi: 10.1371/journal.pgen.1006762 (PMC5428976; doi:10.1371/journal.pgen.1006762)
Supplement: S7 Table — (PDF) [file pgen.1006762.s018.pdf]

S7 Table. qPCR values

**Figure 1E-P**		
**Target**	**Sample**	**Fold Change**
GSTA1	Control	1.15 ± 0.25
GSTA1	WDR23 Isoform 1 o/e	0.62 ± 0.10
GSTA1	WDR23 Isoform 2 o/e	0.35 ± 0.11
CYP3A4	Control	1.07 ± 0.16
CYP3A4	WDR23 Isoform 1 o/e	0.57 ± 0.14
CYP3A4	WDR23 Isoform 2 o/e	0.54 ± 0.14
ACADL	Control	1.00 ± 0.04
ACADL	WDR23 Isoform 1 o/e	0.81 ± 0.04
ACADL	WDR23 Isoform 2 o/e	0.77 ± 0.07
ACADM	Control	1.02 ± 0.08
ACADM	WDR23 Isoform 1 o/e	1.10 ± 0.05
ACADM	WDR23 Isoform 2 o/e	0.87 ± 0.04
GSR	Control	1.09 ± 0.19
GSR	Control + tBHQ	1.80 ± 0.25
GSR	WDR23 Isoform 1 o/e	0.94 ± 0.07
GSR	WDR23 Isoform 1 o/e + tBHQ	1.14 ± 0.07
GSR	WDR23 Isoform 2 o/e	0.99 ± 0.07
GSR	WDR23 Isoform 2 o/e + tBHQ	1.16 ± 0.08
CYP1A1	Control	1.02 ± 0.07
CYP1A1	Control + tBHQ	1.28 ± 0.13
CYP1A1	WDR23 Isoform 1 o/e	1.07 ± 0.12
CYP1A1	WDR23 Isoform 1 o/e + tBHQ	1.09 ± 0.11
CYP1A1	WDR23 Isoform 2 o/e	0.59 ± 0.09
CYP1A1	WDR23 Isoform 2 o/e + tBHQ	0.83 ± 0.04
CPT1A1	Control	1.05 ± 0.12
CPT1A1	Control + tBHQ	1.40 ± 0.11
CPT1A1	WDR23 Isoform 1 o/e	1.06 ± 0.12
CPT1A1	WDR23 Isoform 1 o/e + tBHQ	0.74 ± 0.04
CPT1A1	WDR23 Isoform 2 o/e	1.04 ± 0.13
CPT1A1	WDR23 Isoform 2 o/e + tBHQ	0.47 ± 0.05
ACADS	Control	1.10 ± 0.15
ACADS	Control + tBHQ	1.64 ± 0.15
ACADS	WDR23 Isoform 1 o/e	1.05 ± 0.12
ACADS	WDR23 Isoform 1 o/e + tBHQ	1.02 ± 0.25
ACADS	WDR23 Isoform 2 o/e	1.12 ± 0.20
ACADS	WDR23 Isoform 2 o/e + tBHQ	0.89 ± 0.25
ACADL	Control	1.00 ± 0.04
ACADL	Control + tBHQ	1.14 ± 0.06
ACADL	WDR23 Isoform 1 o/e	0.81 ± 0.04
ACADL	WDR23 Isoform 1 o/e + tBHQ	0.77 ± 0.04
ACADL	WDR23 Isoform 2 o/e	0.77 ± 0.07
ACADL	WDR23 Isoform 2 o/e + tBHQ	0.80 ± 0.05
GCLM	C57BL/6	1.03 ± 0.11
GCLM	*WDR23* KO	1.33 ± 0.04
NQO1	C57BL/6	1.03 ± 0.12

NQO1	*WDR23* KO	1.47 ± 0.05
GSR	C57BL/6	1.00 ± 0.009
GSR	*WDR23* KO	1.49 ± 0.02
**Figure 3D-F**		
**Target**	**Sample**	**Fold Change**
gcs-1	WT (N2)	1.04 ± 0.22
gcs-1	Q80Stop	8.89 ± 1.39
gcs-1	D387N	2.07 ± 0.31
gcs-1	T400I	5.38 ± 0.23
gcs-1	W399Stop	5.91 ± 1.47
gcs-1	Frameshift	5.97 ± 0.28
gcs-1	H310Y	2.38 ± 0.13
gcs-1	D313N	2.37 ± 0.28
gcs-1	G460R	1.66 ± 0.01
gst-4	WT (N2)	1.00 ± 0.09
gst-4	Q80Stop	146.78 ± 25.06
gst-4	D387N	14.36 ± 1.20
gst-4	T400I	66.22 ± 6.15
gst-4	W399Stop	61.44 ± 23.15
gst-4	Frameshift	70.30 ± 1.47
gst-4	H310Y	29.33 ± 1.65
gst-4	D313N	53.83 ± 2.73
gst-4	G460R	13.44 ± 0.58
ugt-11	WT (N2)	1.00 ± 0.04
ugt-11	Q80Stop	5.26 ± 0.86
ugt-11	D387N	4.10 ± 0.52
ugt-11	T400I	4.83 ± 1.03
ugt-11	W399Stop	15.38 ± 1.76
ugt-11	Frameshift	9.72 ± 1.44
ugt-11	H310Y	6.03 ± 0.81
ugt-11	D313N	1.71 ± 0.20
ugt-11	G460R	4. 01 ± 0.35
**Figure 4D**		
**Target**	**Sample**	**Fold Change**
GCLC	Control	0.90 ± 0.06
GCLC	*KEAP1* RNAi	3.59 ± 1.26
GCLC	WDR23 Isoform 1 o/e	0.92 ± 0.02
GCLC	*KEAP1* RNAi + WDR23 Isoform 1 o/e	1.16 ± 0.09
GCLC	WDR23 Isoform 2 o/e	0.97 ± 0.03
GCLC	*KEAP1* RNAi + WDR23 Isoform 2 o/e	1.02 ± 0.05
**Figure 6D-E**		
**Target**	**Sample**	**Fold Change**
GSTA1	Control	1.06 ± 0.12
GSTA1	WDR23 Isoform 1 o/e	0.60 ± 0.13
GSTA1	WDR23 Isoform 2 o/e	0.67 ± 0.14
PRDX1	Control	1.03 ± 0.07
PRDX1	WDR23 Isoform 1 o/e	1.27 ± 0.16
PRDX1	WDR23 Isoform 2 o/e	0.82 ± 0.05
**Figure S2C-Q**		

**Target**	**Sample**	**Fold Change**
GCLM	Control	1.05 ± 0.12
GCLM	WDR23 Isoform 1 o/e	1.05 ± 0.12
GCLM	WDR23 Isoform 2 o/e	0.81 ± 0.22
ABCC1	Control	1.01 ± 0.05
ABCC1	WDR23 Isoform 1 o/e	1.14 ± 0.12
ABCC1	WDR23 Isoform 2 o/e	1.03 ± 0.17
CYP4A11	Control	1.24 ± 0.26
CYP4A11	WDR23 Isoform 1 o/e	0.94 ± 0.14
CYP4A11	WDR23 Isoform 2 o/e	0.79 ± 0.33
PRDX1	Control	1.03 ± 0.10
PRDX1	Control + tBHQ	1.31 ± 0.06
PRDX1	WDR23 Isoform 1 o/e	1.02 ± 0.04
PRDX1	WDR23 Isoform 1 o/e + tBHQ	1.17 ± 0.16
PRDX1	WDR23 Isoform 2 o/e	0.94 ± 0.03
PRDX1	WDR23 Isoform 2 o/e + tBHQ	0.98 ± 0.13
NQO1	Control	1.08 ± 0.14
NQO1	Control + tBHQ	3.72 ± 0.26
NQO1	WDR23 Isoform 1 o/e	1.06 ± 0.12
NQO1	WDR23 Isoform 1 o/e + tBHQ	3.00 ± 0.30
NQO1	WDR23 Isoform 2 o/e	0.81 ± 0.26
NQO1	WDR23 Isoform 2 o/e + tBHQ	4.05 ± 0.58
HO-1	Control	1.18 ± 0.30
HO-1	Control + tBHQ	8.93 ± 1.30
HO-1	WDR23 Isoform 1 o/e	1.30 ± 0.18
HO-1	WDR23 Isoform 1 o/e + tBHQ	13.67 ± 3.96
HO-1	WDR23 Isoform 2 o/e	1.09 ± 0.26
HO-1	WDR23 Isoform 2 o/e + tBHQ	8.05 ± 1.54
KEAP1	Control	1.09 ± 0.16
KEAP1	Control + tBHQ	1.73 ± 0.36
KEAP1	WDR23 Isoform 1 o/e	1.08 ± 0.32
KEAP1	WDR23 Isoform 1 o/e + tBHQ	1.28 ± 0.11
KEAP1	WDR23 Isoform 2 o/e	0.79 ± 0.26
KEAP1	WDR23 Isoform 2 o/e + tBHQ	1.24 ± 0.25
KEAP1	C57BL/6	1.01 ± 0.10
KEAP1	*WDR23* KO	1.33 ± 0.13
SLBP	Control	1.00 ± 0.21
SLBP	*SLBP* RNAi	0.54 ± 0.09
SLBP	Control	1.00 ± 0.26
SLBP	*SLBP* RNAi	0.34 ± 0.40
NRF2	Control	1.00 ± 0.15
NRF2	*SLBP* RNAi	0.78 ± 0.02
HO-1	Control	1.00 ± 0.09
HO-1	*SLBP* RNAi	1.41 ± 0.30
ACADL	Control	1.00 ± 0.31
ACADL	*SLBP* RNAi	0.64 ± 0.33
GCLC	Control	1.00 ± 0.28
GCLC	*SLBP* RNAi	0.80 ± 0.13
NQO1	Control	1.00 ± 0.21

NQO1	*SLBP* RNAi	0.60 ± 0.11
**Figure S6A**		
**Target**	**Sample**	**Fold Change**
wdr-23	WT (N2)	1.00 ± 0.08
wdr-23	Q80Stop	2.53 ± 0.15
wdr-23	D387N	1.79 ± 0.08
wdr-23	T400I	3.72 ± 0.32
wdr-23	W399Stop	2.49 ± 0.85
wdr-23	Frameshift	3.65 ± 0.20
wdr-23	H310Y	2.00 ± 0.10
wdr-23	D313N	1.85 ± 0.12
wdr-23	G460R	1.64 ± 0.11
**Figure S9A**		
**Target**	**Sample**	**Fold Change**
NQO1	Control	1.00 ± 0.05
NQO1	*KEAP1* RNAi	1.76 ± 0.15
NQO1	WDR23 Isoform 1 o/e	0.93 ± 0.03
NQO1	*KEAP1* RNAi + WDR23 Isoform 1 o/e	1.94 ± 0.12
NQO1	WDR23 Isoform 2 o/e	1.03 ± 0.04
NQO1	*KEAP1* RNAi + WDR23 Isoform 2 o/e	1.82 ± 0.16
