# Supplementary material for: WDR23 regulates NRF2 independently of KEAP1
Source: PLoS Genet. 2017 Apr 28;13(4):e1006762. doi: 10.1371/journal.pgen.1006762 (PMC5428976; doi:10.1371/journal.pgen.1006762)
Supplement: S8 Table — (PDF) [file pgen.1006762.s019.pdf]

S8 Table. Luciferase assay values

Figure 1D	
**Sample**	**RLU**
Control	1.00 ± 0.03
WDR23 Isoform 1 o/e	0.75 ± 0.02
WDR23 Isoform 2 o/e	0.73 ± 0.02
**Figure 4C**	
**Sample**	**RLU**
Control	1.00 ± 0.03
KEAP1 RNAi	1.77 ± 0.11
WDR23 Isoform 1 o/e	0.75 ± 0.02
KEAP1 RNAi + WDR23 Isoform 1 o/e	1.06 ± 0.05
WDR23 Isoform 2 o/e	0.73 ± 0.02
KEAP1 RNAi + WDR23 Isoform 2 o/e	0.89 ± 0.03
**Figure S2A-B**	
**Sample**	**RLU**
Control	1.00 ± 0.09
Ce WDR-23 Isoform 1 o/e	0.97 ± 0.07
Ce WDR-23 Isoform 2 o/e	1.07 ± 0.04
Control	1.00 ± 0.09
KEAP1 RNAi	1.34 ± 0.09
Ce WDR-23 Isoform 1 o/e	0.97 ± 0.07
KEAP1 RNAi + Ce WDR-23 Isoform 1 o/e	1.28 ± 0.08
Ce WDR-23 Isoform 2 o/e	1.07 ± 0.04
KEAP1 RNAi + Ce WDR-23 Isoform 2 o/e	1.24 ± 0.05
