# Supplementary material for: WDR23 regulates NRF2 independently of KEAP1
Source: PLoS Genet. 2017 Apr 28;13(4):e1006762. doi: 10.1371/journal.pgen.1006762 (PMC5428976; doi:10.1371/journal.pgen.1006762)
Supplement: S9 Table — (PDF) [file pgen.1006762.s020.pdf]

S9 Table. Immunostaining values

**Figure 6C**		
**Sample**	**% of population**	**# of cells (n)**
Control Diffuse #1	16	21
Control Nuclear #1	84	114
Control Diffuse #2	14	22
Control Nuclear #2	86	133
Control Diffuse #3	18	35
Control Nuclear #3	82	165
WDR23 Isoform 1 o/e Diffuse #1	59	10
WDR23 Isoform 1 o/e Nuclear #1	41	7
WDR23 Isoform 1 o/e Diffuse #2	76	22
WDR23 Isoform 1 o/e Nuclear #2	24	7
WDR23 Isoform 1 o/e Diffuse #3	82	23
WDR23 Isoform 1 o/e Nuclear #3	18	5
WDR23 Isoform 2 o/e Diffuse #1	70	14
WDR23 Isoform 2 o/e Nuclear #1	30	6
WDR23 Isoform 2 o/e Diffuse #2	44	11
WDR23 Isoform 2 o/e Nuclear #2	56	14
WDR23 Isoform 2 o/e Diffuse #3	72	28
WDR23 Isoform 2 o/e Nuclear #3	28	11
**Figure S11G**		
**Sample**	**% of population**	**# of cells (n)**
Control Diffuse #1	12	4
Control Nuclear #1	88	30
Control Diffuse #2	9	9
Control Nuclear #2	91	80
Control Diffuse #3	4	2
Control Nuclear #3	96	54
Control Diffuse #4	7	3
Control Nuclear #4	93	43
WDR23 Isoform 1 o/e Diffuse #1	53	23
WDR23 Isoform 1 o/e Nuclear #1	47	20
WDR23 Isoform 1 o/e Diffuse #2	31	31
WDR23 Isoform 1 o/e Nuclear #2	69	67
WDR23 Isoform 1 o/e Diffuse #3	50	7
WDR23 Isoform 1 o/e Nuclear #3	50	7
WDR23 Isoform 1 o/e Diffuse #4	67	22
WDR23 Isoform 1 o/e Nuclear #4	33	11
WDR23 Isoform 2 o/e Diffuse #1	61	11
WDR23 Isoform 2 o/e Nuclear #1	39	18
WDR23 Isoform 2 o/e Diffuse #2	49	23
WDR23 Isoform 2 o/e Nuclear #2	51	24
WDR23 Isoform 2 o/e Diffuse #3	70	19
WDR23 Isoform 2 o/e Nuclear #3	30	8
WDR23 Isoform 2 o/e Diffuse #4	67	18
WDR23 Isoform 2 o/e Nuclear #4	33	9
